# Single station seismic observations enable high resolution localization of tectonic tremor sources using a vision transformer

**DOI:** 10.1038/s41598-026-58641-5

**Published:** 2026-06-20

**Authors:** Amane Sugii, Yoshihiro Hiramatsu

**Affiliations:** 1 Graduate School of Natural Science and Technology, Kanazawa University, Kanazawa, 920-1192 Japan; 2 Faculty of Geosciences and Civil Engineering, Institute of Science and Engineering, Kanazawa University, Kanazawa, 920-1192 Japan

**Keywords:** Slow earthquakes, Nankai subduction zone, Deep learning, Attention mechanism, Natural hazards, Solid Earth sciences

## Abstract

Precisely locating tectonic tremor events is essential for understanding subduction zone dynamics. However, the lack of impulsive phase arrivals typically necessitates multi-station observations, posing a fundamental challenge for high-resolution monitoring using limited seismic records. Here, we show that single-station three-component wavefields can encode sufficient information to constrain tremor source locations using TremorViT, a vision transformer-based framework designed to estimate three-dimensional coordinates together with aleatoric uncertainties. Applied to the Nankai subduction zone, TremorViT achieves a mean epicentral error of 4.8 km using a single station, which improves to 2.5 km when independent station-level estimates are integrated. Using this approach, we localized 68,101 tremor events between January and September 2016, corresponding to approximately 1135.0 h of cumulative tremor activity. These metrics are substantially larger than those reported in existing network-based catalogs. This discrepancy likely reflects differences in detection sensitivity, event definition, and temporal windowing, even as the model preserves previously documented spatiotemporal patterns. This framework enables high-resolution tremor detection and offers a more granular view of tectonic activity, including potential signals associated with geodetically undetected slow slip events.

**Supplementary Information:**

The online version contains supplementary material available at https://doi.org/10.1038/s41598-026-58641-5.

## Introduction

Tectonic tremor is a distinct class of slow earthquake characterized by slip velocities significantly lower than those of regular earthquakes. Since its initial identification in the Nankai^1^ and Cascadia^2^ subduction zones, tremor has been observed in diverse tectonic settings, including transform faults^3–5^. These events frequently accompany short-duration slow slip events (SSEs), collectively termed Episodic Tremor and Slip^2^, which are considered to play a critical role in the nucleation of megathrust earthquakes^6,7^. Monitoring the spatiotemporal evolution of tremor events is therefore essential for constraining the frictional properties of plate interfaces and improving our understanding of seismogenic processes.

Accurate tremor source localization remains inherently challenging. Low signal-to-noise ratios and the absence of impulsive phase arrivals limit the applicability of conventional earthquake location techniques. Existing approaches can be broadly divided into two categories: waveform-similarity methods, such as envelope cross-correlation^1^, and template matching based on low-frequency earthquakes (LFEs)^8^, and methods incorporating additional physical or statistical constraints, including amplitude and radiated energy^9^, and maximum likelihood estimation^10^. While these methods have achieved considerable success, they exploit only part of the available waveform information, limiting their ability to fully capture the complexity of tremor signals.

Single-station approaches offer a promising alternative. Although tremor consists of overlapping LFEs without distinct phases, recent studies have suggested that single-station wavefields contain rich information for source characterization. Various features, including nonlinear statistical dependencies^11^, frequency-band energy ratios^12^, relationships between seismic energy rate and moment rate^13^, and temporal evolution of spectral characteristics^14^, have been shown to capture aspects of tremor signals related to source properties. These findings suggest that single-station waveforms may contain sufficient information for source characterization; however, directly constraining source locations from such data remains challenging and has not been fully established.

Deep learning (DL) provides a natural framework for integrating such complex features from three-component waveforms. Recent DL-based studies have successfully classified tremor, noise, and earthquakes using spectrogram-based architectures^15–17^, while others have detected LFEs based on waveform characteristics^18,19^. In addition, DL regression models have demonstrated the capability to estimate hypocenters for regular earthquakes^20,21^. Although some studies have improved tremor monitoring by combining DL detection with conventional post-processing approaches, most existing methods either focus on classification tasks or rely on multi-station observations for source location^17,22^. As a result, direct estimation of tremor source locations from single-station data remains largely underexplored.

In this study, we propose a DL framework, TremorViT, designed to locate tremor sources directly from single-station three-component waveform data. Our model integrates a convolutional neural network (CNN)-based feature extractor with a vision transformer (ViT)^23^ to predict three-dimensional (3D) coordinates together with their associated aleatoric uncertainties. By leveraging the frequent occurrence of tremor and the dense seismic network coverage of the Nankai subduction zone for training and validation, we demonstrate that high-resolution tremor monitoring is achievable at the single-station level. This approach provides a robust framework for better characterizing tectonic tremor sources through the integration of location estimates from multiple stations.

## Dataset

### Overview

For consistency, we refer to the CNN used for waveform classification as TremorDetector and the ViT-based single-station source location model as TremorViT.

**Fig. 1:**
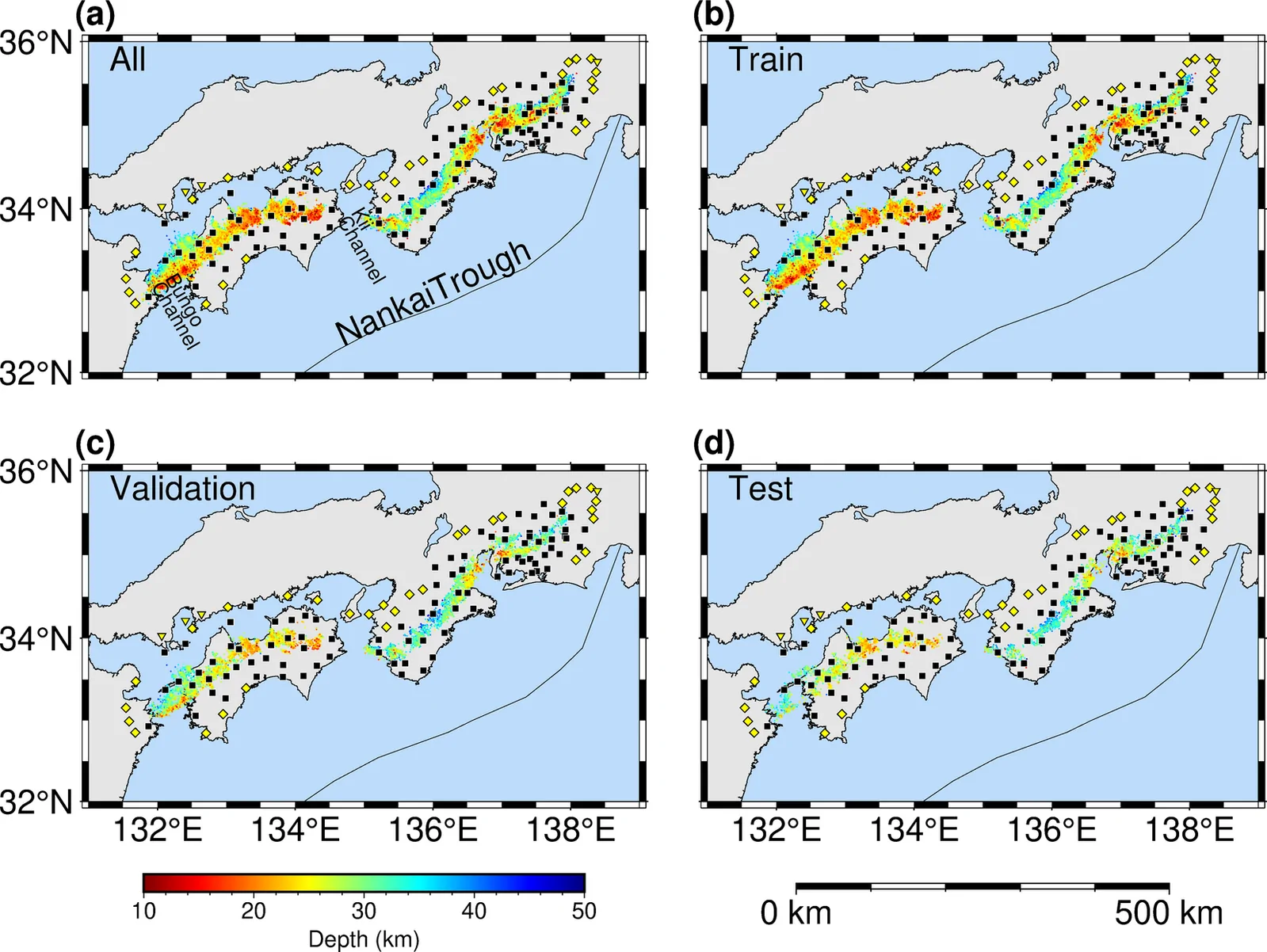
Map of seismic stations and tectonic tremor epicenters. (**a**) All tremor events used in this study, and the (**b**) training, (**c**) validation, and (**d**) test datasets. Black squares, yellow diamonds, and inverted yellow triangles represent stations included in the analysis, those excluded based on data-availability criteria, and those for which no waveform segments with tremor probability ≥ 0.9 were identified, respectively. The maps were generated using GMT 6.6 (https://www.generic-mapping-tools.org).

The dataset comprises three-component velocity waveforms recorded at 129 Hi-net stations operated by the National Research Institute for Earth Science and Disaster Resilience (NIED) (Fig. 1). We analyzed one-minute waveform segments sampled at 100 Hz from January 2008 to September 2016. Tremor origin times and hypocenter locations were obtained from the catalog of Mizuno and Ide^10^, which was constructed using a modified envelope correlation method. Events outside the primary Nankai tremor zone, such as those in active volcanic regions, were removed to focus exclusively on tectonic tremor.

Because tectonic tremor is generally characterized by long-lasting and nearly continuous signals, defining the onset and termination of individual events is inherently ambiguous. In this study, following common practice in previous studies^16,17,24^, tremor activity was represented using one-minute waveform windows. Each window was treated as an analysis unit, providing a practical balance between capturing sustained tremor energy and preserving temporal resolution for source tracking.

### Dataset for TremorDetector

To ensure reliable event identification, we adopted TremorDetector^22^, a CNN-based model trained to classify waveforms into tremor, earthquake, and noise. Tremor samples were selected from high-activity periods (≥ 5 events per hour) and were visually verified; any time windows contaminated by P-wave arrivals from regular earthquakes at stations within 40 km of a tremor epicenter were excluded. Earthquake samples were filtered by magnitude and depth to exclude low-amplitude or ambiguous signals (*M* < 1.0 for depths of < 50 km; *M* < 3.0 for greater depths), and noise samples were extracted from periods without cataloged activity.

Following the input preparation of Sugii et al.^16^, the three-component waveforms were bandpass filtered (2–8 Hz) and converted into 52 × 52 pixel spectrograms (0–10 Hz). The dataset comprised 104,091 spectrograms per category (tremor, noise, and earthquake) for training/validation (2008–2015) and 22,480 per category for testing (January–September 2016)^22^.

### Dataset for TremorViT

For each Hi-net station, we extracted all cataloged tremor events^10^ within a 50 km epicentral distance. Theoretical S-wave arrival times were calculated assuming a constant S-wave velocity of 3.5 km/s^9^, and one-minutes waveform windows were centered on these arrival times.

These windows were evaluated using the pre-trained TremorDetector, and only event-station pairs with tremor probabilities ≥ 0.9 were retained. As a result of this filtering, four stations were excluded. This criterion ensured high-confidence training samples while preserving waveform diversity. Events associated with fewer than three stations were excluded. Following this refinement, we excluded stations with fewer than 80 test samples in 2016 or fewer than 600 total samples across the study period, leaving 94 stations for primary model training and evaluation (Supplementary Table S1). The remaining 31 stations were reserved as an independent dataset to assess regional model generalization.

The refined catalog was partitioned chronologically into training (2008–2014), validation (2015), and test (January–September 2016) subsets. The final dataset comprised 641,718 training, 89,386 validation, and 56,744 test waveforms (Fig. 1). During training, we applied random temporal perturbations of ± 20 s to the window centers to prevent the model from over-relying on the signal’s central position for source location estimation. Window centers remained fixed for validation and testing.

## Results

### Performance evaluation on the test dataset

We evaluated TremorViT using the 2016 test dataset, treating hypocenters from the Mizuno and Ide^10^ catalog as catalog-based reference labels, since the true tremor locations are inherently unknown. The difference between the predicted probability distribution center and the reference hypocenter is defined as the epicentral or absolute depth error (ADE).

**Fig. 2:**
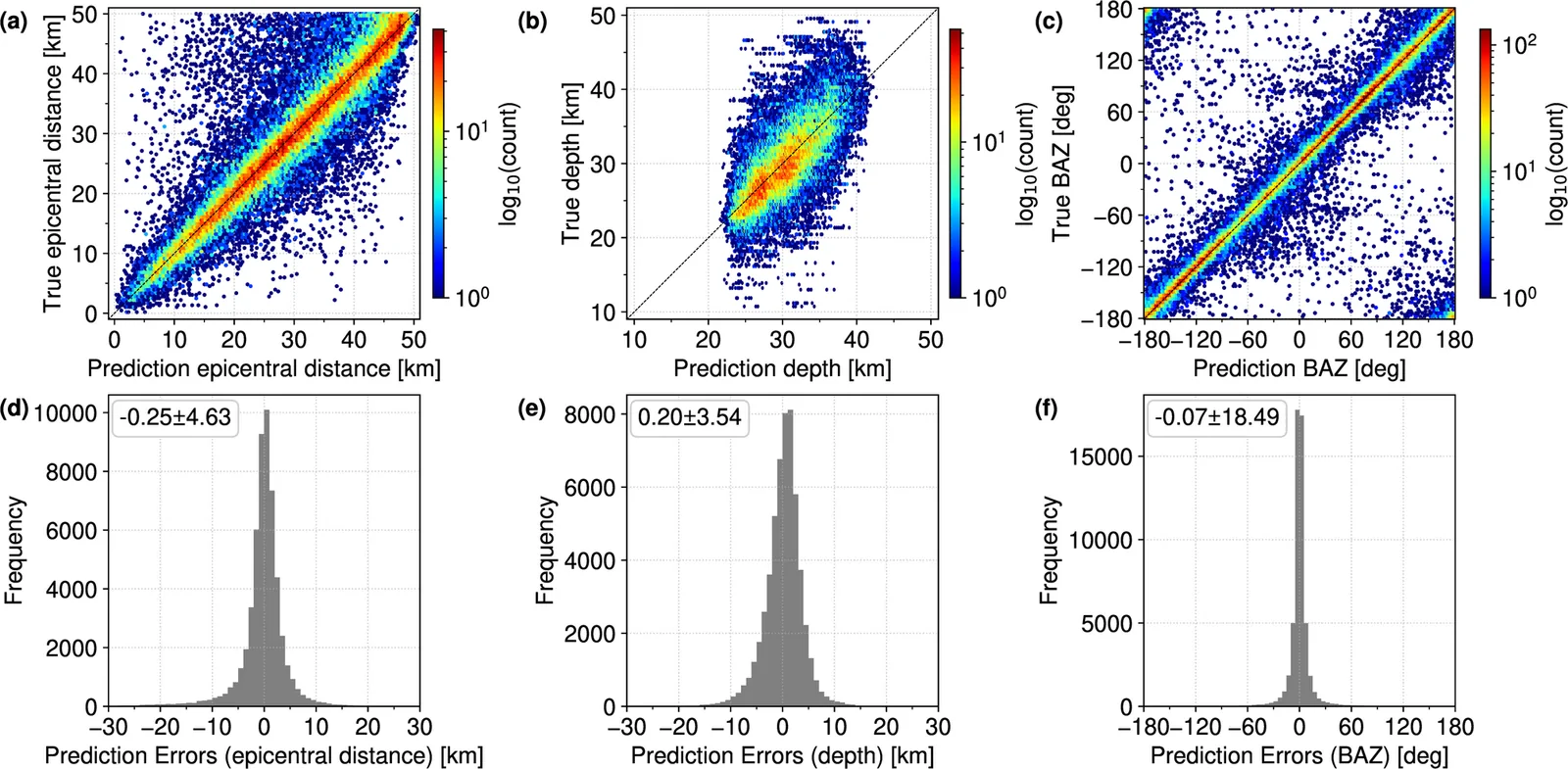
Comparison of predicted values and catalog-based reference values. (**a**–**c**) Relationships between predicted and reference values, and (**d–f**) frequency distributions of prediction errors for (**a**,**d**) epicentral distance, (**b**,**e**) depth, and (**c**,**f**) back-azimuth. In the upper panels, colors indicate the density of data points, and the black lines represent 1:1 correspondence. In the lower panels, the mean and standard deviation are shown in the upper-left corners.

For single-station predictions (*n* = 56,744), we report both the mean and median to account for skewed distribution. The epicentral error yielded a mean of 4.75 ± 7.31 km (mean ± s.d.; median 2.66 km; 90th percentile 9.42 km), comparable to the approximately 3 km horizontal errors reported for regular earthquakes using envelope cross-correlation^25^. The ADE exhibited a mean of 2.59 ± 2.41 km (mean ± s.d.; median 1.94 km; 90th percentile 5.54 km). Predicted and reference epicentral distances and back-azimuths were strongly concentrated along the 1:1 reference line (Fig. 2), whereas source depth showed weaker correlation.

To test whether the model learned physical waveform features rather than spatial priors (i.e., the geographical distribution of the training data), we compared predictions with a baseline defined by the mean hypocenter location of the training data for each station. Because the error distributions were non-normal, we used a one-tailed binomial test to evaluate whether the proportion of samples in which TremorViT produced smaller localization error than the baseline exceeded the null-hypothesis value of 0.5. The model outperformed the baseline in 93% of samples for both epicentral and hypocentral errors (*n* = 56,744, *p*-value < 10^−16^; Supplementary Fig. S1).

For source depth, 59% of samples improved over the baseline (*n* = 56,744, *p*-value < 10^−16^), indicating weaker depth constraints. Random ± 20-s perturbations to the window centers resulted in negligible error increases (mean epicenter error 4.81 ± 7.39 km, mean ADE 2.60 ± 2.40 km; mean ± s.d.), demonstrating robustness to arrival-time uncertainties.

### Uncertainty as a metric for reliability and discrimination

TremorViT outputs a three-dimensional Gaussian distribution, enabling the quantification of aleatoric (data-dependent) uncertainty. We used the volume of the 95% confidence ellipsoid (km^3^) as the uncertainty metric. Tremor samples predominantly yielded volumes on the order of 1 × 10^3^ km^3^, corresponding to an isotropic-equivalent confidence radius of approximately 6.2 km (Supplementary Fig. S2). To evaluate whether the aleatoric uncertainty reflects actual performance, we examined its correlation with localization errors using Spearman’s rank correlation coefficient ($\:\rho\:$), owing to the non-normal distributions. Epicentral error showed a substantial positive correlation ($\:\rho\:$ = 0.62, *n* = 56,744, *p*-value < 10^− 16^), indicating that the larger predicted uncertainties corresponded to larger actual errors. In contrast, the correlation with ADE was considerably weaker ($\:\rho\:$ = 0.18, *n* = 56,744, *p*-value < 10^− 16^), consistent with horizontal locations being better constrained than source depth.

**Fig. 3:**
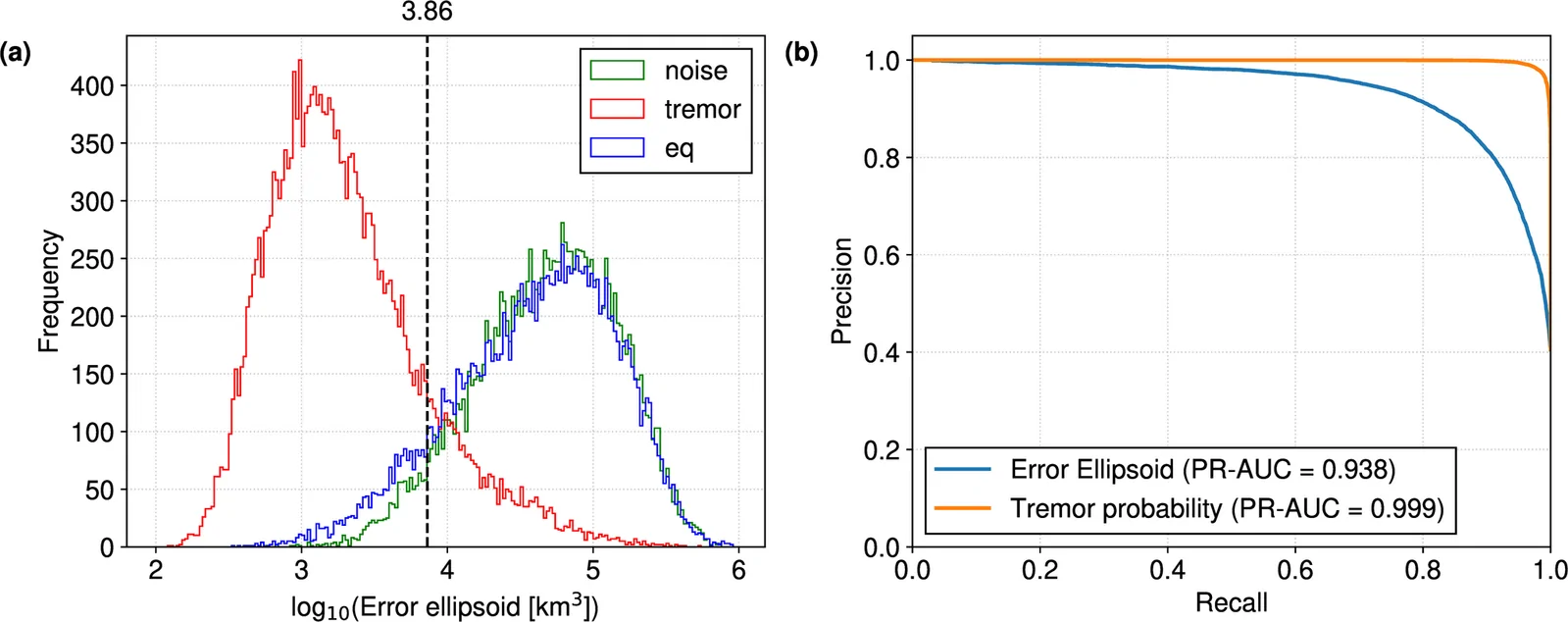
Comparison of uncertainties among tremor, noise, and earthquake waveforms. (**a**) Frequency distributions of aleatoric uncertainty volumes for tremor, noise, and earthquake signals. The dashed vertical line denotes the uncertainty threshold that maximizes the F1-score for tremor detection. (**b**) Precision–recall curves for tremor detection obtained by thresholding the aleatoric uncertainty volume, compared with those from the TremorDetector.

Remarkably, using the TremorDetector evaluation dataset (tremor: *N* = 22,378; noise: *N* = 16,165; earthquake: *N* = 16,678), TremorViT’s aleatoric uncertainty effectively discriminated tremor from non-tremor signals, despite no explicit classification training. In our dataset, tremor waveforms generally exhibited significantly smaller uncertainty volumes (~ 1 × 10^3^ km^3^) than noise or earthquakes (~ 1 × 10^5^ km^3^) (Fig. 3). Thresholding uncertainty volumes yielded a maximum F1-score of 0.865, reflecting a strong balance between precision and recall, with a precision–recall area under the curve (PR-AUC) of 0.938. Although slightly below TremorDetector performance, this indicates that the model’s internal confidence provides a robust proxy for signal validity. Earthquake and noise signals also exhibited distinct uncertainty distributions across stations (Supplementary Fig. S3), suggesting that the model recognizes waveforms inconsistent with the learned tremor-source dynamics.

### Continuous monitoring and multi-station integration

**Fig. 4:**
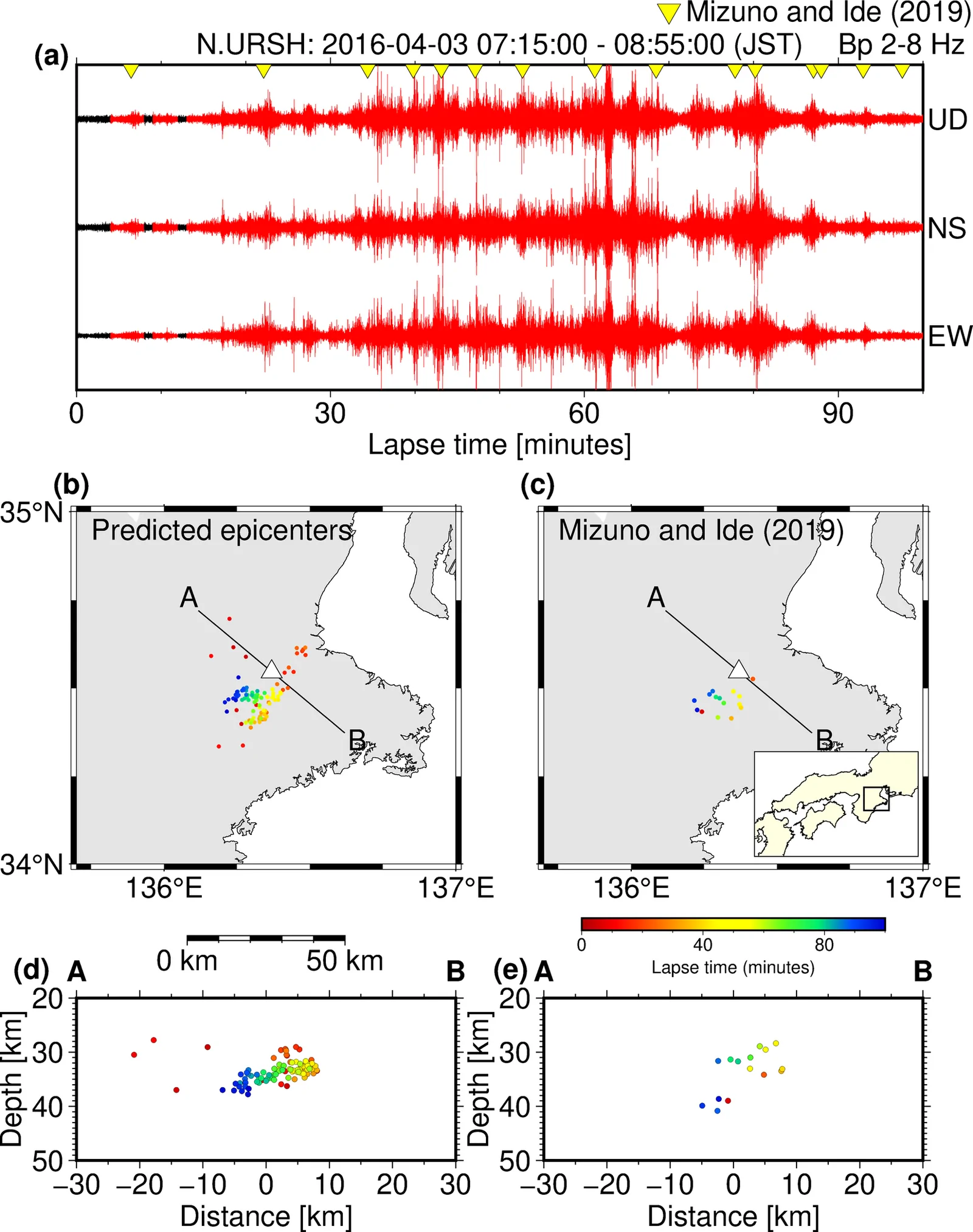
Example application of TremorViT to continuous waveform data at station N.URSH. (**a**) Bandpass-filtered (2–8 Hz) three-component velocity waveforms starting at 07:15 JST on 3 April 2016. Yellow inverted triangles indicate tremor events archived in the Mizuno and Ide^10^ catalog, and red portions of the waveforms denote tremor detections identified by TremorDetector (probability ≥ 0.9). (**b**,**c**) Map views of tremor epicenters predicted by (**b**) TremorViT and (**c**) those reported in the Mizuno and Ide^10^ catalog. Line A–B indicates the location of the vertical cross-sections shown in (**d**,**e**). (**d**,**e**) Depth distributions along profile A–B for (**d**) TremorViT predictions and (**e**) the Mizuno and Ide^10^ catalog. In all spatial plots, symbol colors represent lapse time from the start of the window, and the white triangle marks the station location.

Continuous monitoring was performed by first identifying tremor waveforms using TremorDetector (tremor probability ≥ 0.9) and subsequently determining their source locations via TremorViT. Applying this workflow to 100 min of continuous data at station N.URSH (starting at 07:15 JST on 3 April 2016) revealed 95 tremor events, representing a six-fold increase over the 15 events listed in the existing catalog (Fig. 4). Predicted locations exhibited high spatiotemporal coherence, consistent with visual inspections of tremor-like signals not documented in the catalog. Similar robust results were obtained across different stations and regions (Supplementary Figs. S4, S5), demonstrating that the framework captures a more granular view of tremor activity than traditional network-based methods.

By integrating independent station-level predictions through a six-step procedure (1, detection; 2, uncertainty-based pruning; 3, pairwise spatial validation; 4, graph-based clustering; 5, covariance intersection and 6, multiple-source discrimination; see Methods), we further enhanced location accuracy. For the test dataset, this integrated approach successfully localized for 6139 events, yielding a mean epicentral error and a mean ADE of 2.50 ± 2.34 km and 2.42 ± 2.30 km, respectively (mean ± s.d.). The uncertainty-based pruning (step 2) rejected 10 events, and the *k*-core filtering (step 4) removed 165 events, predominantly in station-sparse areas such as Bungo Channel and Kii Channel (Supplementary Fig. S6) where the 50-km epicentral distance constraint limited the number of qualifying stations. In three specific time windows, two spatially distinct hypocenter candidates separated by approximately 17–36 km were temporarily retained after covariance-intersection integration (see Discussion for details). These competing solutions were subsequently evaluated using the multiple-source discrimination step described in Methods.

Applying this framework to continuous data at one-minute intervals from 1 January to 30 September 2016 identified 68,956 tremor events. Each source location was determined by integrating source estimates from multiple stations, with an average of 6.5 stations providing mutually consistent location and uncertainty results per window. Events isolated more than 5 km in epicentral distance and more than 12 h in time were excluded from subsequent analyses^24^. The final dataset contained 68,101 events, approximately an order of magnitude more than the reference catalog^10^.

**Fig. 5:**
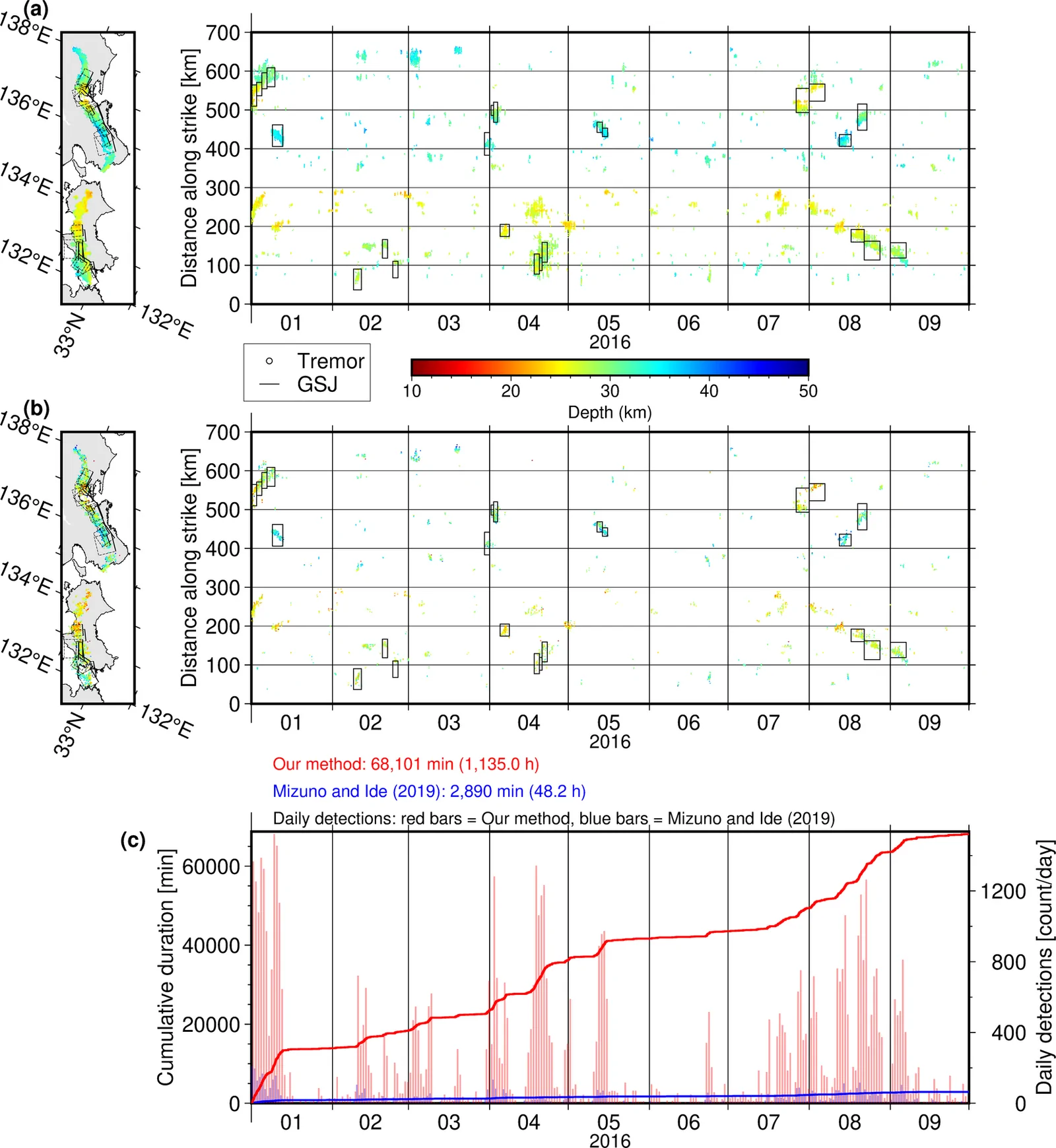
Comparison of the spatiotemporal distributions of tremor epicenters (**a**) detected by the integrated TremorViT framework and (**b**) those archived in the Mizuno and Ide^10^ catalog. Epicentral locations were estimated at one-minute intervals from 1 January to 30 September 2016. Colored dots represent individual tremor events, and open rectangles indicate the spatiotemporal extents of SSEs reported by the Geological Survey of Japan (GSJ)^26,27^. (**c**) Cumulative tremor duration and daily event counts. Red denotes the results obtained by the proposed method, whereas blue denotes those from the Mizuno and Ide^10^ catalog.

Because tremor tends to occur in clustered or quasi-continuous episodes, the number of detected events depends on the event definition and windowing scheme. In this study, source locations were estimated independently for each 1-min window, meaning adjacent windows may represent temporally overlapping or continuous tremor activity rather than distinct physical events. We thus used cumulative duration, defined as the total time of one-minute windows localized as tremors. This approach yielded 1135.0 h of tremor activity, substantially longer than the 48.2 h reported in the reference catalog. However, these two measures are not directly comparable because the reference catalog primarily consists of shorter-duration tremor episodes, typically shorter than one-minute window, identified using different detection and segmentation criteria. The discrepancy, therefore, likely reflects not only an increase in tremor localization sensitivity in this study but also differences in detection thresholds, temporal windowing, and event definitions. Despite differing measures, the spatiotemporal distribution aligns with previously reported patterns (Fig. 5), supporting the physical plausibility of our results.

We evaluated the effect of multi-station integration by comparing its output with the single-station results for the period shown in Fig. 4. Detected events decreased from 95 (station N.URSH) to 83 after integration, mainly because low-amplitude signals lacking spatial consistency were removed. Even after this filtering, the integrated catalog remains substantially larger than the existing catalog. Most additional detections occur during periods of elevated tremor activity, when overlapping signals cause the detection efficiency of conventional envelope-correlation methods to decrease. The newly identified events show no significant differences in amplitude or frequency characteristics from those in the reference catalog (Supplementary Fig. S7), supporting their reliability.

## Discussion

Our findings demonstrate that single-station three-component records contain far more source information than traditionally assumed. TremorViT extracts robust source constraints and uncertainties directly from individual wavefields using transformer-based deep learning, enabling higher-resolution monitoring from minimal observations. The following sections discuss parameter dependence, geophysical implications, and remaining limitations.

### Source constraints and depth sensitivity

Horizontal coordinates are more reliably constrained than depth. This is physically reasonable for tectonic tremor signals; back-azimuth and epicentral distance are likely encoded in first-order waveform features such as horizontal polarization (particle motion) and relative amplitude ratios, whereas depth depends on subtle information. Without distinct, depth-sensitive phase arrivals, depth remains inherently difficult to resolve from single-station data.

The model assigns larger uncertainties to less well-constrained estimates, reflecting prediction reliability. This correlation is strong for epicentral locations but weaker for depth due to sparse depth-sensitive information. Consequently, TremorViT appropriately represents relative confidence without being overconfident, particularly in horizontal components.

Despite limited resolution, the aggregated depth increases systematically from southeast to northwest (Supplementary Fig. S8). This aligns with the results of Mizuno and Ide^10^ (see their Fig. 6b) and the Philippine Sea Plate geometry, suggesting that the model captures large-scale structural trends. This agreement indicates that TremorViT exploits waveform information beyond conventional envelope-based methods.

Although depth accuracy is limited, our primary interpretations rely on the more robustly constrained horizontal distributions. Accordingly, depth estimates are better suited for identifying large-scale structural trends rather than characterizing individual events.

### High-resolution tremor catalogs as complementary geodetic tools

**Fig. 6:**
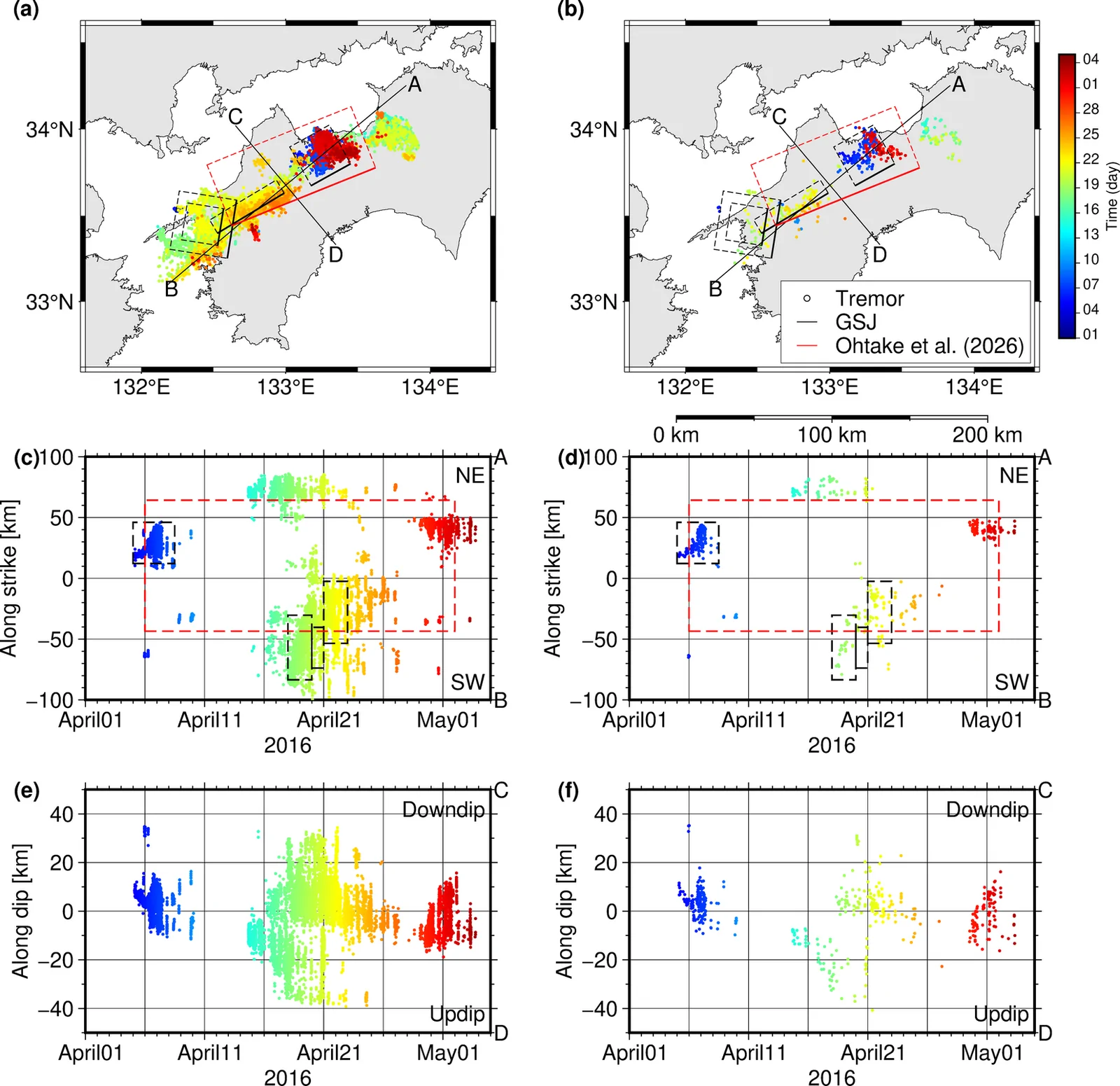
Comparison of the spatiotemporal distributions of tremor epicenters (**a**) detected by the integrated TremorViT framework and (**b**) those archived in the Mizuno and Ide^10^ catalog. Epicentral locations were estimated at one-minute intervals from 1 January to 30 September 2016. Colored dots represent localized one-minute tremor windows. Black open rectangles indicate the spatiotemporal extents of SSEs reported by the Geological Survey of Japan (GSJ)^26,27^ whereas the red open rectangle indicates the SSE reported by Ohtake et al.^28^. (**c**,** d**) Spatiotemporal evolution along strike (profile A–B). (**e**,**f**) Cross-sectional views along the dip direction (profile C–D). In (**c**–**d**), black dotted rectangles correspond to the GSJ SSEs and the red dotted rectangle corresponds to the SSE reported by Ohtake et al. as shown in (**a**,**b**). In all panels, the color gradient represents the temporal progression of tremor activity.

Our framework produced high-resolution tremor catalogs that reveal transient fault-slip processes in detail. In western Shikoku (from 1 April to 5 May 2016), we identified 10,334 events, a 22-fold increase over the reference catalog (477 events) (Fig. 6). Correspondingly, cumulative tremor duration, defined as the total time of one-minute windows with localized tremor sources, increased from 3.7 h to 172.2 h (47-fold). Despite differing from the reference catalog’s energy-based definition, this consistency in temporal occurrence enables clearer visualization of spatiotemporal behaviors (Supplementary Fig. S9).

Comparison with four SSEs (*M*_w_ 5.4–5.9) reported by the Geological Survey of Japan (GSJ)^26,27^ showed tremor clusters that corresponded spatiotemporally to these events (Fig. 6c). We also resolved rapid tremor increases near the Bungo Channel following the *M*_w_ 7.0 Kumamoto earthquake, supporting the interpretation that dynamic stress perturbations promoted slow-slip activity^28^. Fine-scale clustering further suggests that some larger SSEs may consist of sequences of smaller sub-events, providing complementary constraints for geodetic slip inversion.

Dense tremor clusters in areas lacking geodetic signals (Fig. 5) may indicate undetected SSEs, suggesting that TremorViT can supplement geodetic networks limited by detection thresholds or station coverage. Integrating such catalogs into slip-inversion models could improve subduction zone energy budgets by including previously unresolved seismic and aseismic energy releases.

### Interpretability and the attention mechanism

The ViT architecture dynamically weights waveform segments through self-attention, enabling the model to capture long-range temporal dependencies and cross-component relationships. Attention rollout^29^ shows that TremorViT preferentially emphasizes tremor-like waveform segments rather than background noise (Supplementary Fig. S10). Masking experiments, in which three-component waveform segments were replaced with zeros over 3 s windows shifted every 0.6 s, showed that suppressing visible tremor packets in the 2–8 Hz band causes larger degradation in location accuracy (Supplementary Fig. S11).

These results suggest that the model uses physically meaningful waveform characteristics, such as coherent tremor energy, polarization patterns, and relative amplitude variations, that contain source-direction and distance information.

Nevertheless, with approximately six million trainable parameters, the physical basis of the learned representations remains only partly understood. Possible cues include polarization, amplitude ratios, scattering characteristics, or subtle temporal correlations, but these require explicit testing. Improving interpretability and linking model decisions to measurable physical processes remains important future goals.

### Limitations and future perspectives

Although TremorViT enables high-resolution tremor monitoring, broader application still faces several limitations.

#### Station and path dependencies

Performance decreased at stations excluded from training. For 18,479 samples from such stations, the mean epicentral error increased to 35.07 ± 18.34 km (mean ± s.d.; median 32.48 km), approximately an order of magnitude larger than that observed at trained stations. The mean ADE rose to 5.05 ± 3.68 km (mean ± s.d.). This suggests that TremorViT likely learns station-specific waveform representations influenced by path and site effects. Although this limits direct transferability without fine-tuning, it also indicates that single-station records contain richer source information than previously exploited.

#### Adaptation to new stations

We fine-tuned the model using the three excluded stations with the largest numbers of samples. For each station, 50 samples were randomly selected for validation and 500 for testing, with the remaining data used for training. Fine-tuning with 50–500 training samples substantially improved performance (Supplementary Fig. S12). In particular, 400 training samples markedly reduced localization errors, indicating that the pre-trained model can be efficiently adapted to new stations. Further robustness may come from unsupervised preprocessing that separates tremor-like signals from noise or extracts station-invariant waveform features.

#### Geometric ambiguity and multi-source resolution

Our framework yielded three instances where covariance-intersection produced dual hypocenter candidates separated by 17–36 km (Supplementary Figs. S13–S15). These bifurcations may reflect simultaneous sources or geometric ambiguity from single-station constraints. To resolve this, we applied a discrimination step based on TremorViT’s 50-km training range: if stations from another cluster fell within 50 km of a source, only the solution with the smaller uncertainty was retained. While this procedure suppresses mutually dependent or geometrically redundant solutions, it does not rule out genuine simultaneous activity. Attention rollout showed no systematic differences between competing solutions, suggesting the ambiguity stems from station geometry rather than waveform features. Nonetheless, these dual-source instances imply that some tremor episodes involve more complex spatiotemporal behavior than a single-source model can capture.

#### Sensitivity to reference catalog quality

Reported location errors reflect discrepancies relative to the reference catalog rather than absolute accuracy. Uncertainties in the reference labels can introduce spatially variable bias, particularly in station-sparse areas like the Bungo and Kii Channels. In these regions, strict spatial consistency criteria (e.g., k-core filtering) reject more events to avoid overinterpreting poorly constrained solutions. Consequently, our framework provides conservative estimates and may underrepresent activity where station coverage is limited.

#### Dependence on input window length

The one-minute input window is a practical compromise between signal content and temporal resolution. The model retained reasonable localization performance even when parts of the waveform were masked, implying that useful information is not uniformly distributed across the full window (Supplementary Fig. S16). Shorter time windows may therefore be feasible, but reduced waveform duration increased predictive uncertainty, indicating a trade-off between temporal resolution and stability.

### Methods

### Architecture of the convolutional neural network (TremorDetector)

To extract reliable tremor signals, we used a CNN that estimates the probability that a one-minute-long, three-component spectrogram corresponds to a tremor, earthquake, or noise signal. The CNN architecture, adapted from Sugii et al.^16^, consists of three convolutional layers (3 × 3 kernels), two max-pooling layers, two dropout layers, and a 32-unit fully connected layer, followed by a three-unit SoftMax output layer. Rectified linear unit activation was applied to all hidden layers.

Training was performed with a batch size of 64 and a learning rate of 1 × 10^−4^ using the Adam optimizer^30^, minimizing the categorical cross-entropy loss. We stopped training early if the validation loss failed to improve for four consecutive epochs, with a maximum limit of 500 epochs. The model achieving the lowest validation loss was selected for subsequent analysis. Further details regarding the model architecture, training procedure, and performance metrics are provided in Jinde et al.^22^.

### Architecture and training of the vision transformer model (TremorViT)

**Fig. 7:**
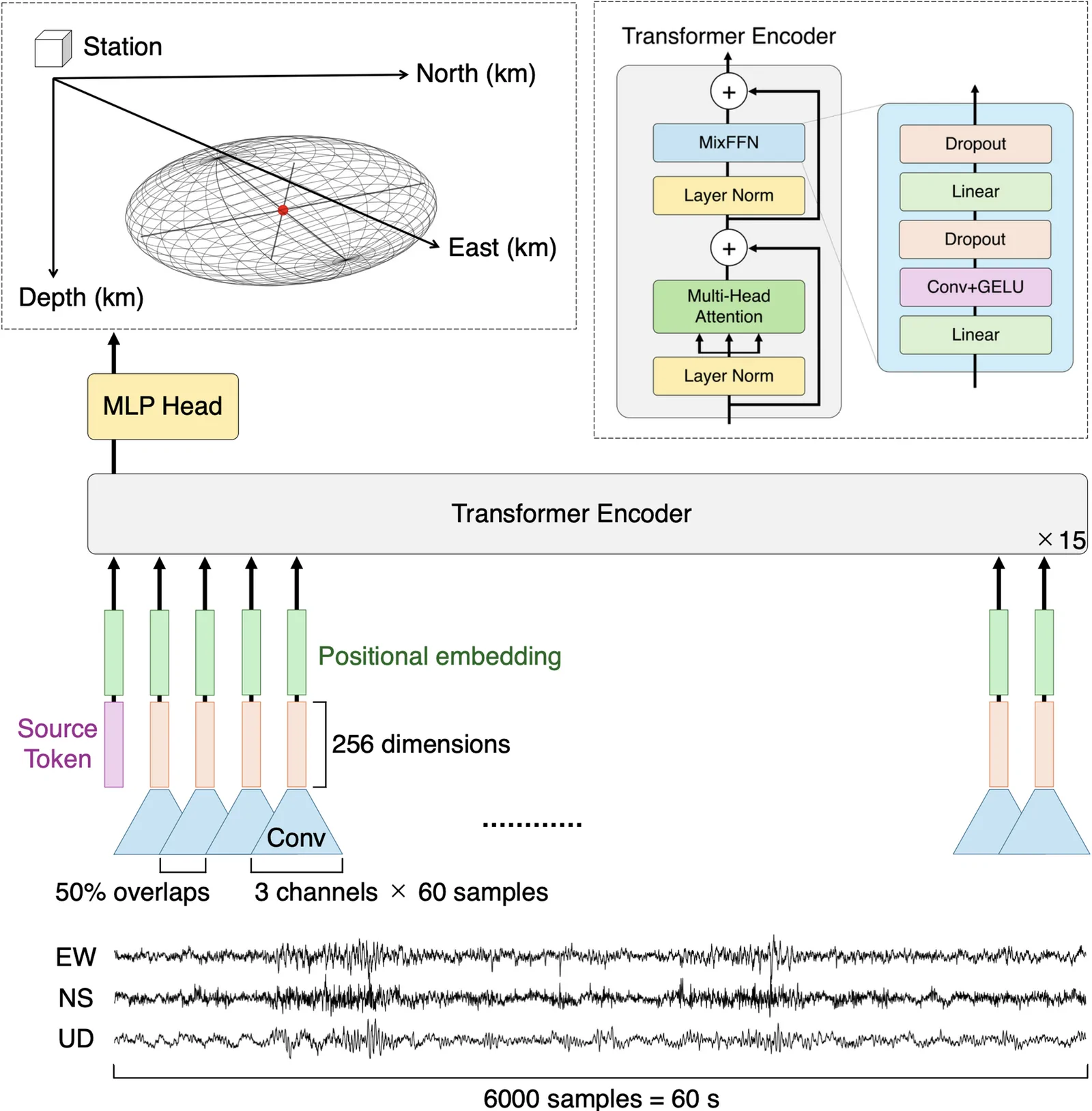
Architecture of the vision transformer (ViT) model for source estimation. The input waveform is tokenized into fixed-length segments (orange blocks) and processed by a stack of Transformer layers. The upper right dashed box details the internal structure of a Transformer layer, which integrates multi-head self-attention with a feed-forward network augmented by a one-dimensional convolution (Conv). MLP denotes a multilayer perceptron. The upper left dashed box provides a schematic of the model output, representing the predicted parameters of a three-dimensional Gaussian distribution for source localization.

To estimate tremor source localization from single-station data, we developed TremorViT, an architecture based on ViT^23^ (Fig. 7). Transformers employ a self-attention mechanism that models relationships among all elements in an input sequence^31^.

The input consists of one-minute-long, three-component velocity waveforms (6000 samples × 3 components), which are divided into partially overlapping 0.6-s segments (60 samples × 3 components). A convolutional layer (kernel size 60, stride 30) embeds these segments into patch tokens; this specific stride setting was found to yield superior performance compared to alternative configurations while maintaining a reasonable computational cost. An auxiliary “source token” is appended to this sequence to aggregate spatial information across the entire waveform duration and learnable positional encodings are added to incorporate temporal order.

The sequence is processed through 15 Transformer layers equipped with multi-head self-attention. In each layer, the feed-forward network (FFN) is augmented by a one-dimensional convolutional layer inserted between two point-wise linear projections, following linear–convolution–activation–linear sequence. This architecture introduces a local inductive bias to the model while retaining the global receptive field of the standard FFN structure^32,33^. The output corresponding to the source token is passed to a multilayer perceptron head to predict the parameters of a three-dimensional Gaussian distribution: the relative north–south distance, east–west distance, and depth, along with the full covariance matrix $\:\boldsymbol{\Sigma\:}$. This probabilistic formulation enables simultaneous estimation of the source location and its associated aleatoric uncertainty. Relative locations are defined in a local Cartesian coordinate system centered at the station (positive for north, east, and downward).

The model was trained by minimizing the negative log-likelihood (NLL):

1 $$\:\begin{array}{c}NLL=-log\left(\frac{1}{{\left(2\pi\:\right)}^{\frac{3}{2}}{\left|\boldsymbol{\Sigma\:}\right|}^{\frac{1}{2}}}\mathrm{e}\mathrm{x}\mathrm{p}\left(-\frac{1}{2}{\left(\varDelta\:\boldsymbol{\mu\:}-\varDelta\:\boldsymbol{x}\right)}^{\mathrm{T}}{\boldsymbol{\Sigma\:}}^{-1}\left(\varDelta\:\boldsymbol{\mu\:}-\varDelta\:\boldsymbol{x}\right)\right)\right) \end{array}$$

where $\:\varDelta\:\boldsymbol{x}$ is the reference relative position vector and $\:\varDelta\:\boldsymbol{\mu\:}$ is the predicted mean position vector. We used the schedule-free RAdam optimizer^34^ with a batch size of 64. Among several tested learning rates (4 × 10^− 4^, 5 × 10^− 4^, and 6 × 10^− 4^), 4 × 10^− 4^ was selected for achieving the lowest validation loss. Early stopping was applied after 10 consecutive epochs without improvement. A complete list of hyperparameters is provided in Supplementary Table S2.

To ensure a valid covariance matrix, we parameterized the predicted $\:\boldsymbol{\Sigma\:}\:$ via its Cholesky factor **L** (a lower triangular matrix) such that $\:\boldsymbol{\Sigma\:}=\mathbf{L}{\mathbf{L}}^{\mathrm{T}}$. The model outputs the mean vector and the six independent elements of $\:\mathbf{L}$ ($\:{\mathbf{L}}_{11},\:{\mathbf{L}}_{22},{\mathbf{L}}_{33},{\mathbf{L}}_{21},{\mathbf{L}}_{31},{\mathbf{L}}_{32}$). We enforced $\:{\mathbf{L}}_{ii}>0$ by applying a softplus function to the diagonal terms, ensuring that $\:\boldsymbol{\Sigma\:}$ is positive definite.

### Integration of multi-station predictions

We integrated the independent station-level estimates into a consistent tremor source location using a six-step procedure:

Step 1: Station-level detection and source estimation.

Segments with tremor probabilities ≥ 0.9 (from TremorDetector) were processed by TremorViT.

Step 2: Uncertainty-based pruning.

Each station-level prediction was filtered based on its associated aleatoric uncertainty, quantified by the covariance ellipsoid volume. Only estimates with uncertainties ≤ 2 × 7281 km^3^ (twice the threshold optimized in Fig. 3) were retained, effectively pruning low-confidence predictions.

Step 3: Pairwise spatial validation.

The pairwise Mahalanobis distance (*D*_M_) between Gaussian distributions *P* and *Q* was computed:

2 $$\:\begin{array}{c}{D}_{\mathrm{M}}^{2}\left(P,\:Q\right)={\left({\boldsymbol{\mu\:}}_{P}-{\boldsymbol{\mu\:}}_{Q}\right)}^{{{\rm\:T}}}{\left({\boldsymbol{\Sigma\:}}_{P}+{\boldsymbol{\Sigma\:}}_{Q}\right)}^{-1}\left({\boldsymbol{\mu\:}}_{P}-{\boldsymbol{\mu\:}}_{Q}\right) \end{array}$$

where $\:\boldsymbol{\mu\:}$ and $\:\boldsymbol{\Sigma\:}$ denote the mean vector and covariance matrix, respectively. Pairs with $\:{D}_{\mathrm{M}}^{2}$ > 7.815 (corresponding to a 95% confidence level for a three-degree-of-freedom system) were considered as spatially inconsistent and excluded from further clustering.

Step 4: Graph-based clustering.

We constructed a graph from the remaining station pairs and applied *k*-core decomposition (with *k* = 2)^35^ to identify mutually consistent clusters, allowing for the simultaneous detection of multiple tremor sources.

Step 5: Fusion via covariance intersection (CI).

Source estimates within a cluster were aggregated using the CI method^36^, yielding a conservative fused distribution:

3 $$\:\begin{array}{c}{{\Sigma\:}}_{\mathrm{C}\mathrm{I}}^{-1}=\sum\:_{i=1}^{n}{\omega\:}_{i}{{\Sigma\:}}_{i}^{-1},\:{\begin{array}{c}\:\:\:\:\\\:\:\mu\:\end{array}}_{CI}={{\Sigma\:}}_{\mathrm{C}\mathrm{I}}\sum\:_{i=1}^{n}{\omega\:}_{i}{{\Sigma\:}}_{i}^{-1}{\mu\:}_{i} \end{array}$$

where $\:{\mu\:}_{i}$ and $\:{{\Sigma\:}}_{i}$ are the mean and covariance of the *i*-th station-level estimate, respectively, and $\:{\omega\:}_{i}$ is a non-negative weight satisfying $\:\sum\:_{i=1}^{n}{\omega\:}_{i}=1$. The weights were optimized to minimize the log-determinant of the fused covariance, ensuring a conservative uncertainty estimates without requiring knowledge of inter-station correlations.

Step 6: Multiple-source discrimination.

Although graph-based clustering permits multiple concurrent sources, single-station estimation can produce bifurcated solutions due to geometric ambiguity. To suppress these, we introduced a discrimination step based on TremorViT’s 50-km training range. For each candidate, if stations from another cluster were located within 50 km of the source, the solutions were deemed non-independent; in such cases, only the solution with the smaller uncertainty volume was retained. Conversely, candidates without overlapping stations within the 50-km radius were classified as independent, simultaneous tremor sources.

## Supplementary Information

Below is the link to the electronic supplementary material.

Supplementary Material 1

## Data Availability

We used catalogs reported by the Japan Meteorological Agency. The tectonic tremor catalog can be downloaded from the Slow Earthquake Database^37^ (http://www-solid.eps.s.u-tokyo.ac.jp/~sloweq/). Seismograms recorded by Hi-net were downloaded from the Hi-net website (https://www.hinet.bosai.go.jp). The tremor catalog created in this study and the code of TremorViT’s are available at https://github.com/amanegeophys/TremorViT.
