# Supplementary material for: Single station seismic observations enable high resolution localization of tectonic tremor sources using a vision transformer
Source: Sci Rep. 2026 Jun 20;16:28507. doi: 10.1038/s41598-026-58641-5 (PMC13572398; doi:10.1038/s41598-026-58641-5)
Supplement: Supplementary file 1 [file 41598_2026_58641_MOESM1_ESM.pdf]

Supplementary Materials for

Single station seismic observations enable high resolution localization of tectonic tremor sources using a vision transformer

Amane Sugii[1], Yoshihiro Hiramatsu[2,*]

[1]Graduate School of Natural Science and Technology, Kanazawa University, Kanazawa 920-1192, Japan
[2]Faculty of Geosciences and Civil Engineering, Institute of Science and Engineering, Kanazawa University, Kanazawa 920-1192, Japan
[*]yoshizo@staff.kanazawa-u.ac.jp

This PDF file includes:

Tables S1 toS2
Figures S1 to S16

Table S1. Numbers of training, validation, and test samples for each station used in this study.

Station	Train (2008-2014)	Validation (2015)	Test (2016/01-09)	Longitude	Latitude
N.UWAH	23318	2700	1129	132.5055	33.4270
N.IKTH	19993	2336	465	132.3072	33.4719
N.KWBH	19950	2422	1276	132.7480	33.5022
N.OOZH	19684	2408	1159	132.6011	33.5807
N.HIYH	17613	2193	1031	132.7980	33.3347
N.MISH	17367	1946	509	132.1157	33.3719
N.TBEH	17288	2032	1351	132.8032	33.7112
N.IKKH	15603	1867	1492	133.1444	33.6472
N.SJOH	15231	1962	1509	133.1840	33.8650
N.GHKH	14403	1990	1449	133.3641	33.7079
N.YNDH	14301	1646	1200	133.0016	33.5388
N.TSMH	13403	1062	197	132.5552	33.0556
N.MTYH	13135	1847	1271	133.5760	33.7543
N.SINH	12996	2031	1309	133.6497	33.9154
N.TSYH	11756	1346	879	132.9891	33.2683
N.ASHH	10056	1234	1068	137.4039	35.2194
N.KHUH	9777	1544	863	133.8243	33.6748
N.KSHH	9753	1189	1025	137.4058	35.2608
N.HYSH	9628	1458	1190	136.0294	34.4237
N.MGWH	9614	1676	1158	136.3293	34.3580
N.IKWH	9607	1606	856	133.8932	33.9997
N.URSH	9566	1902	1061	136.3692	34.5470
N.KAWH	9179	1286	1154	136.0022	34.2925
N.ASUH	9178	1113	1021	137.3359	35.1392
N.SADH	9040	1611	797	134.0918	34.0112
N.TBRH	8943	1136	894	133.0658	33.9023
N.STRH	8877	1033	760	137.5350	35.1543
N.KYDH	8648	1502	842	134.1295	33.8777
N.YZEH	8539	1640	898	136.0512	34.6413
N.KRTH	8429	1103	1026	135.8369	34.3061
N.WATH	8326	1614	856	136.5866	34.3532

Table S1. (continued)

Station	Train (2008-2014)	Validation (2015)	Test (2016/01-09)	Longitude	Latitude
N.MNOH	7939	1408	891	133.7081	34.1980
N.AYKH	7908	1319	735	133.9493	34.2186
N.HRYH	7867	918	648	137.6261	35.3095
N.TKEH	7417	1037	766	135.8574	33.9692
N.TYEH	7314	830	566	137.7268	35.1840
N.MASH	7257	1514	689	136.5033	34.5424
N.TKWH	7232	1032	744	135.6489	33.9666
N.TOEH	7114	812	550	137.7208	35.0814
N.HNZH	7018	999	722	135.5369	34.1373
N.OYMH	6723	1524	762	136.2835	34.7662
N.TDEH	6582	841	728	137.4114	34.9512
N.HOUH	6511	760	537	137.6243	35.0002
N.OWSH	6378	869	686	136.1689	34.0637
N.NUKH	6188	803	847	137.2993	34.9189
N.HAZH	6089	855	846	137.1057	34.7884
N.TT2H	6065	702	455	137.8409	35.0086
N.KGIH	5514	788	774	137.0570	35.3059
N.SIKH	5440	475	151	131.8695	32.9278
N.OTOH	5272	796	398	135.5952	33.6948
N.INOH	4917	578	526	133.4388	33.5313
N.MS2H	4863	513	354	137.9181	35.2233
N.OKZH	4807	582	708	137.2377	34.9635
N.MCTH	4546	989	732	136.9385	34.7401
N.SSRH	4529	576	388	137.4876	34.8976
N.HRKH	4491	685	312	135.2122	33.9804
N.MSNH	4408	503	343	137.9267	35.2986
N.ANJH	4286	647	570	137.0567	34.9319
N.NTGH	4105	484	288	137.4706	35.4571
N.MYKH	3740	433	447	133.0581	34.1918
N.NGKH	3662	492	628	137.0474	35.1842
N.YOTH	3615	390	358	137.2464	35.4865

Table S1. (continued)

Station	Train (2008-2014)	Validation (2015)	Test (2016/01-09)	Longitude	Latitude
N.MSKH	3533	277	177	137.9359	35.1981
N.KTDH	3503	585	278	135.4396	33.6894
N.INMH	3480	574	263	135.2176	33.8252
N.SSMH	3400	499	236	135.5454	33.5559
N.MKBH	3201	527	339	137.5109	34.8050
N.NKTH	3012	413	196	135.8619	33.6072
N.AC2H	2892	456	301	137.7346	35.4786
N.HNOH	2861	834	306	136.2610	34.9638
N.TKGH	2784	375	219	137.8767	35.5143
N.KNNH	2668	445	250	134.3156	33.6628
N.KHOH	2613	302	325	135.9969	33.7644
N.YOKH	2385	745	419	136.4563	34.9849
N.GNOH	2324	809	289	136.4245	34.8224
N.TOHH	2319	330	212	132.4046	33.9291
N.SGRH	2153	523	220	136.0316	34.8530
N.MKIH	2052	390	267	134.1482	34.2700
N.KNSH	2024	301	136	132.1040	33.8260
N.IIDH	1991	356	266	138.0058	35.4489
N.HMAH	1908	331	358	136.7002	35.2768
N.THNH	1904	325	188	137.4393	34.7853
N.INSH	1895	245	87	137.6616	34.8343
N.HWSH	1781	318	167	134.4994	33.7751
N.TOKH	1706	320	172	134.5337	33.9902
N.NMKH	1628	289	197	133.3493	34.3785
N.HTNH	1424	158	156	138.2076	35.3029
N.HKWH	1322	130	95	138.1351	35.0966
N.KTGH	1313	222	118	134.1197	33.5421
N.OHCH	1195	228	174	134.3042	34.2264
N.SONH	1082	101	94	138.1972	35.3070
N.KYSH	816	146	188	136.8509	35.2177
N.NGIH	796	130	90	137.6105	35.6056
N.GSIH	755	113	117	133.8192	33.5294

Table S2. TremorViT architecture and training details.

Category	Parameter	Value / Description
Dataset	Training set size	641,718 waveforms (2008–2014)
	Validation set size	89,386 waveforms (2015)
	Test set size	56,744 waveforms (2016/01–09)
Input	Duration	1 minute (100Hz)
	Components (in order)	east-west, north-south, vertical
	Normalization	Z-score normalization (per each component)
Patch Embedding	Kernel size	60
	Stride	30
	Channel Size	256
Transformer	Number of layers	15
	Number of attention heads	8
	Positional embedding	Learnable
Mix-FFN (convolutional feed-forward network)	Kernel size	3
	Dimensions	256
Others	Activation	GeLU
	Normalization	LayerNorm
	Dropout rate	0.1
Token Design	Source token dimensions	256
Output	Prediction target	9 parameters of a trivariate normal distribution
Loss Function	Type	Negative log-likelihood
Optimization	Optimizer	RAdam [1]
	Learning rate	0.0004
	Batch size	64

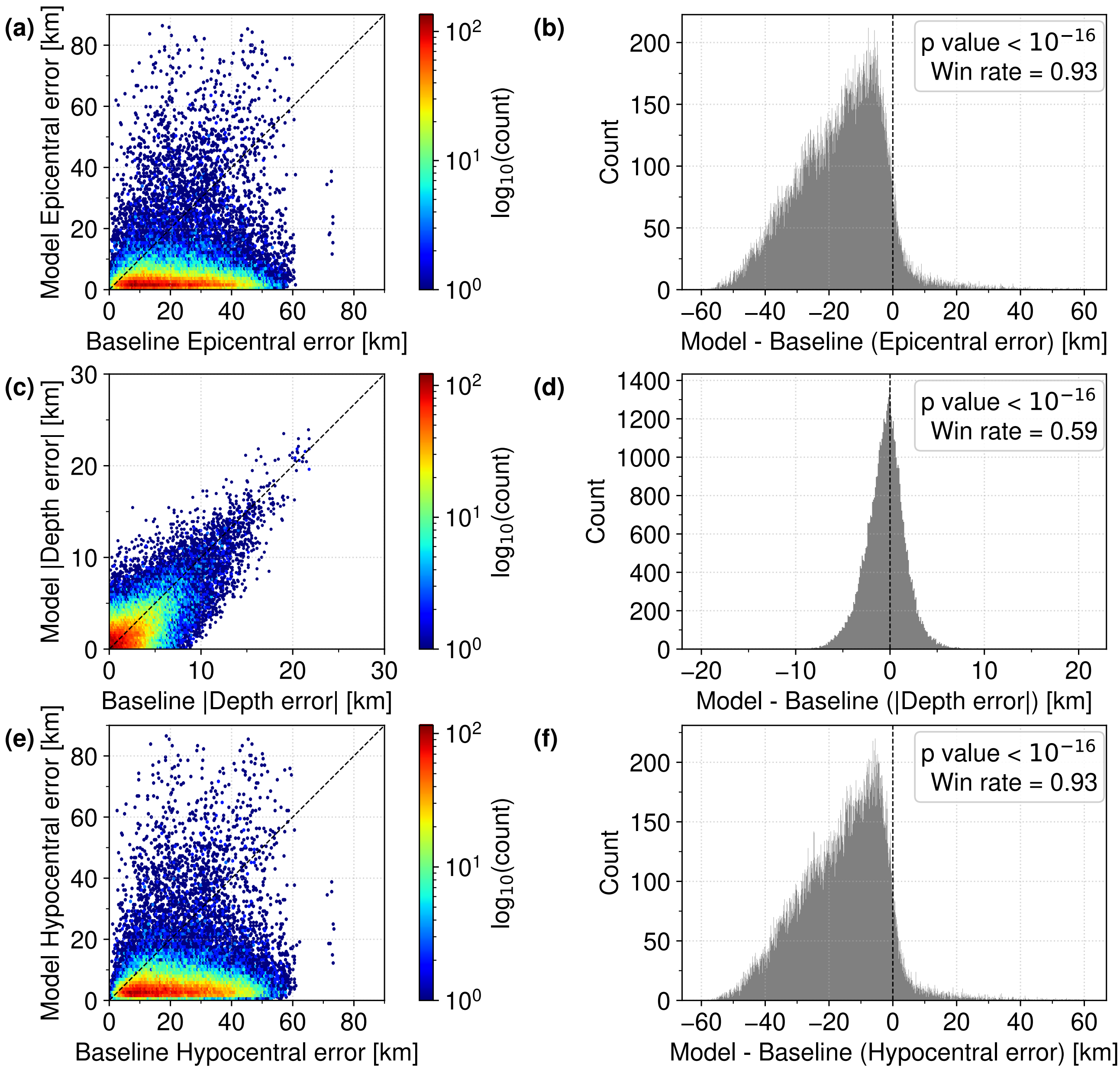

Fig. S1 Results of the baseline test. Comparison of location errors between TremorViT and the baseline model: **(a, b)** epicenter errors, **(c, d)** absolute depth errors, and **(e, f)** hypocentral errors. **(a, c, e)** Direct comparisons, with black dashed lines representing a 1:1 correspondence. **(b, d, f)** Histograms of the error differences (TremorViT – baseline). Upper-right annotations in the histograms indicate *p*-values and the fraction of samples where the TremorViT errors are smaller than the baseline errors ('Win rate').

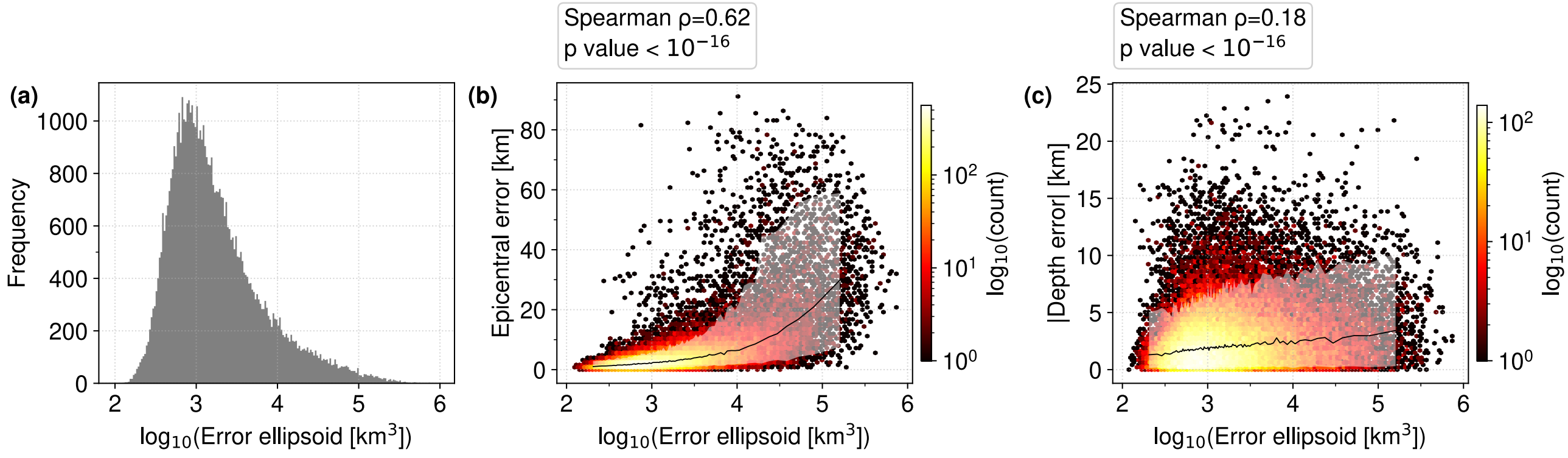

Fig. S2 Relationship between TremorViT uncertainty and location errors. (a) Frequency distribution of predicted aleatoric uncertainty for the test dataset. **(b, c)** Relationships between uncertainty and **(b)** epicentral error and **(c)** absolute depth error. The black lines represent the median errors, and the white shaded regions indicate the 5 th – 95 th percentile range calculated within 100 quantile bins. Upper-left annotations show the Spearman's rank correlation coefficient (ρ) and the corresponding p-value, demonstrating the reliability of the uncertainty estimates as error predictors.

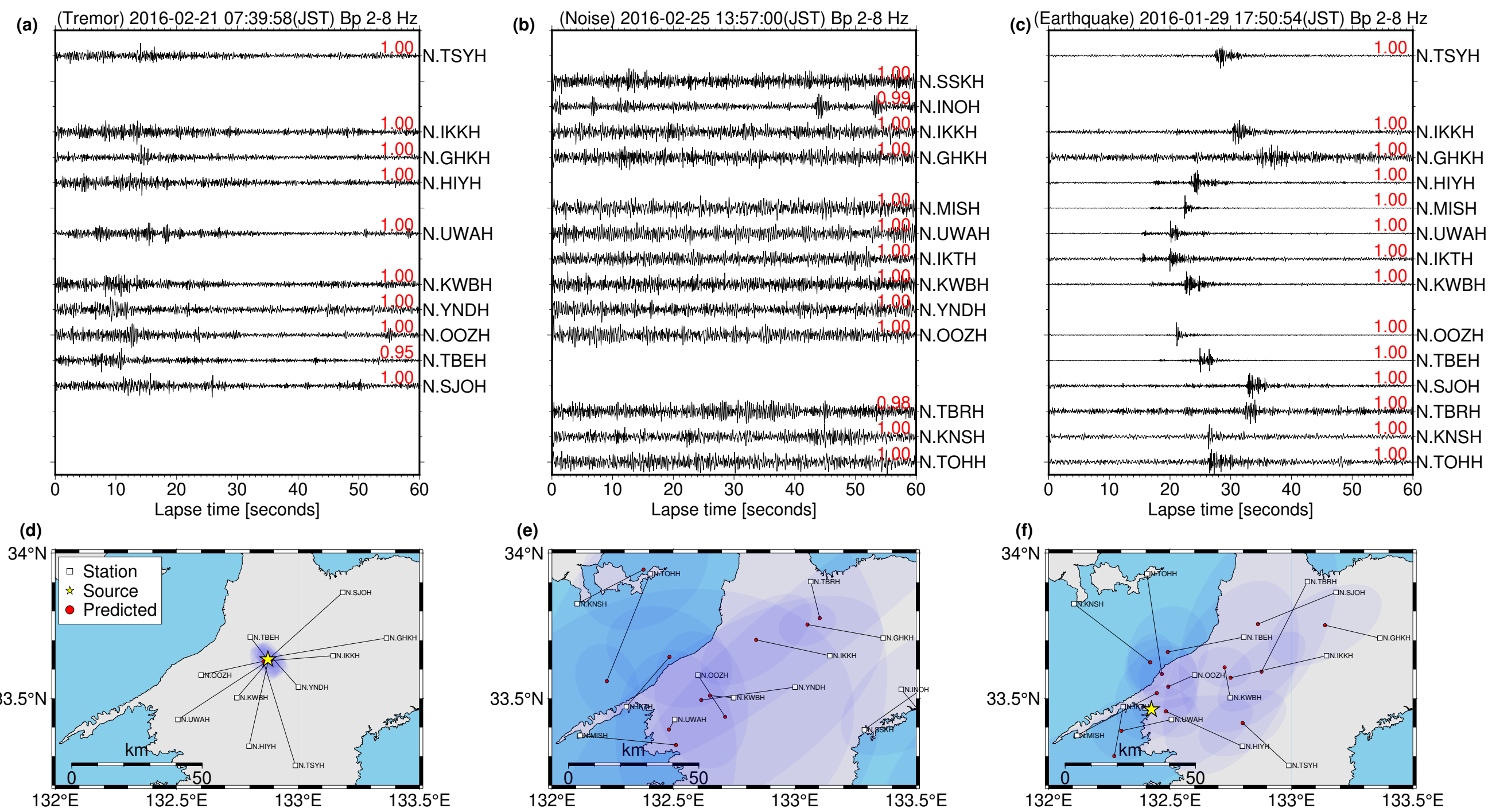

Fig. S3 TremorViT predictions for tremor, noise, and earthquake events. (a–c) East–west (EW) component waveforms for **(a)** tremor, **(b)** noise, and **(c)** earthquake events recorded at individual stations. Overlaid red numbers indicate classification probabilities assigned by TremorDetector for each category. Station codes are labeled to the right of each waveform. **(d–f)** Corresponding epicentral predictions by TremorViT. Red circles represent predicted epicenters and yellow stars denote cataloged locations (for tremor and earthquake events). White squares are station locations, and black lines connect each station to its specific prediction. The blue ellipses represent the 95% confidence intervals derived from the predicted aleatoric uncertainties.

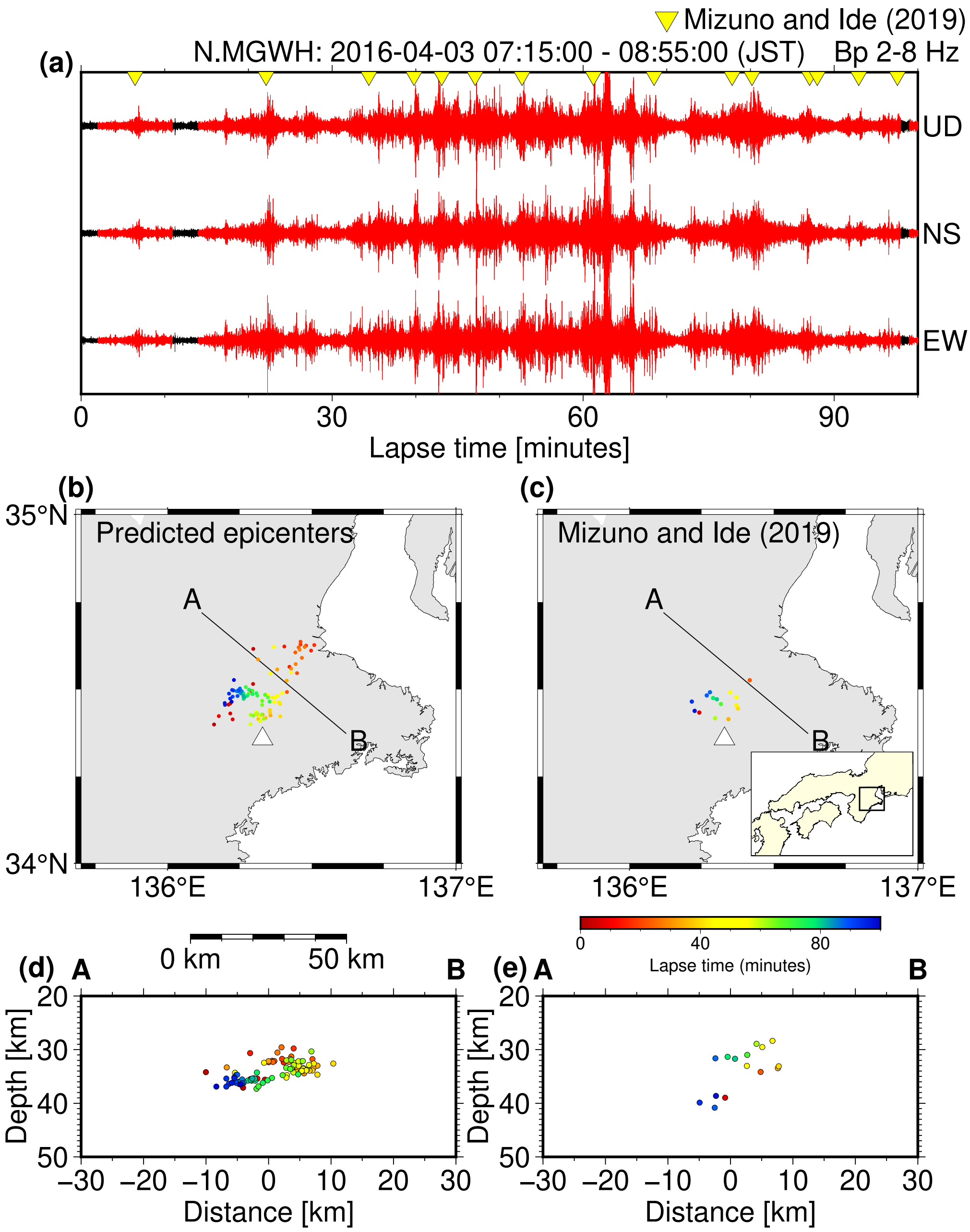

Fig. S4 Example application of TremorViT to continuous waveform data at station N.MGWH. **(a)** Bandpass-filtered (2–8 Hz) three-component velocity waveforms starting at 07:15 JST on 3 April 2016. Yellow inverted triangles indicate tremor events listed in the Mizuno and Ide [2] catalog and red segments denote tremor detections by TremorDetector (probability ≥ 0.9). **(b, c)** Map views of tremor epicenters predicted by **(b)** TremorViT and **(c)** those reported in the Mizuno and Ide [2] catalog. Line A–B indicates the location of the vertical cross-section shown in panels **(d)** and **(e)**. **(d, e)** Depth distributions along profile A–B for **(d)** TremorViT predictions and **(e)** the Mizuno and Ide [2] catalog. In all spatial plots, symbol colors represent lapse time from the start of the window, and the white triangle marks the station location.

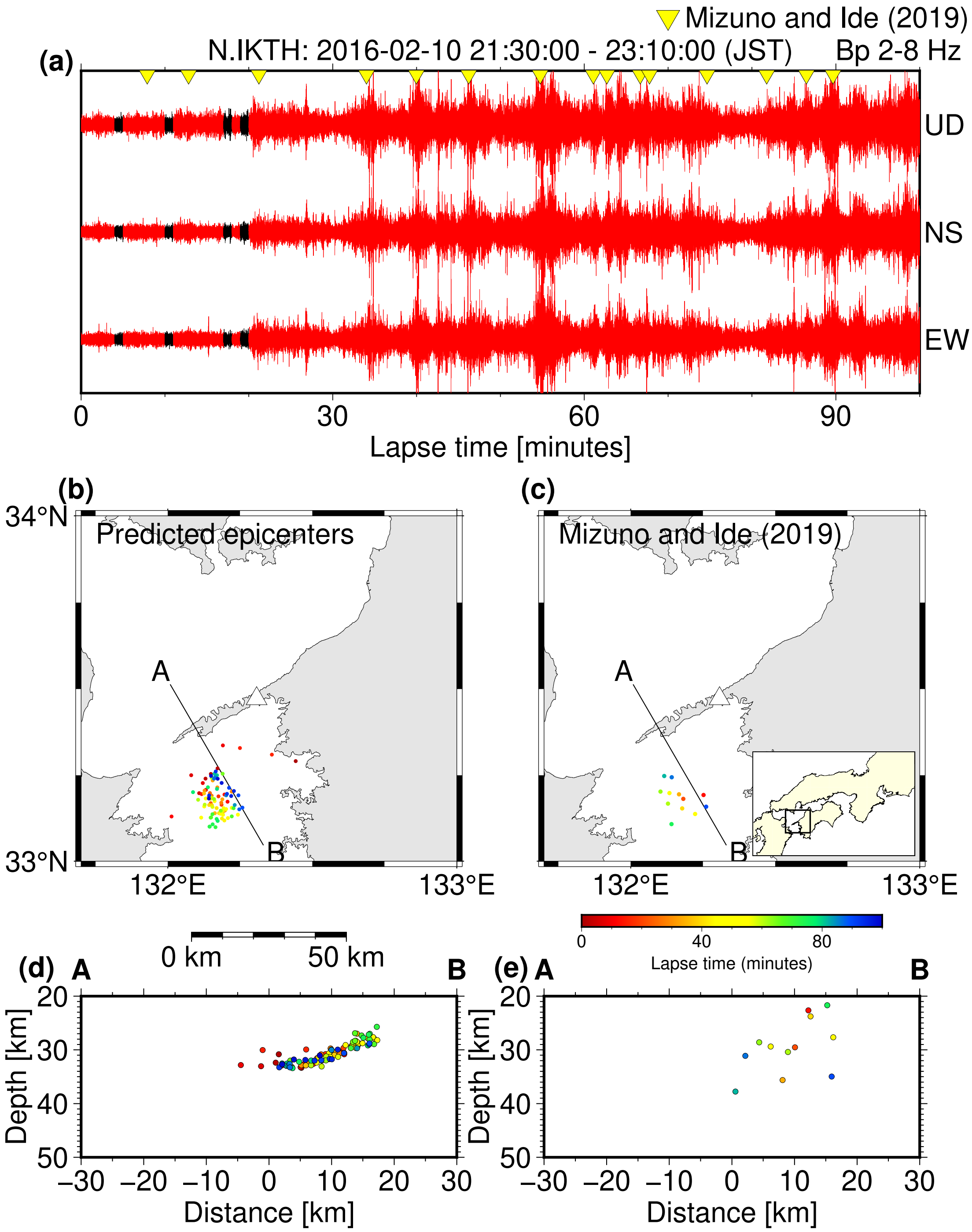

Fig. S5 Example application of TremorViT to continuous waveform data at station N.IKTH. As in Fig. S4, but for station N.IKTH and a different 100-min interval starting at 21:30 JST on 10 February 2016.

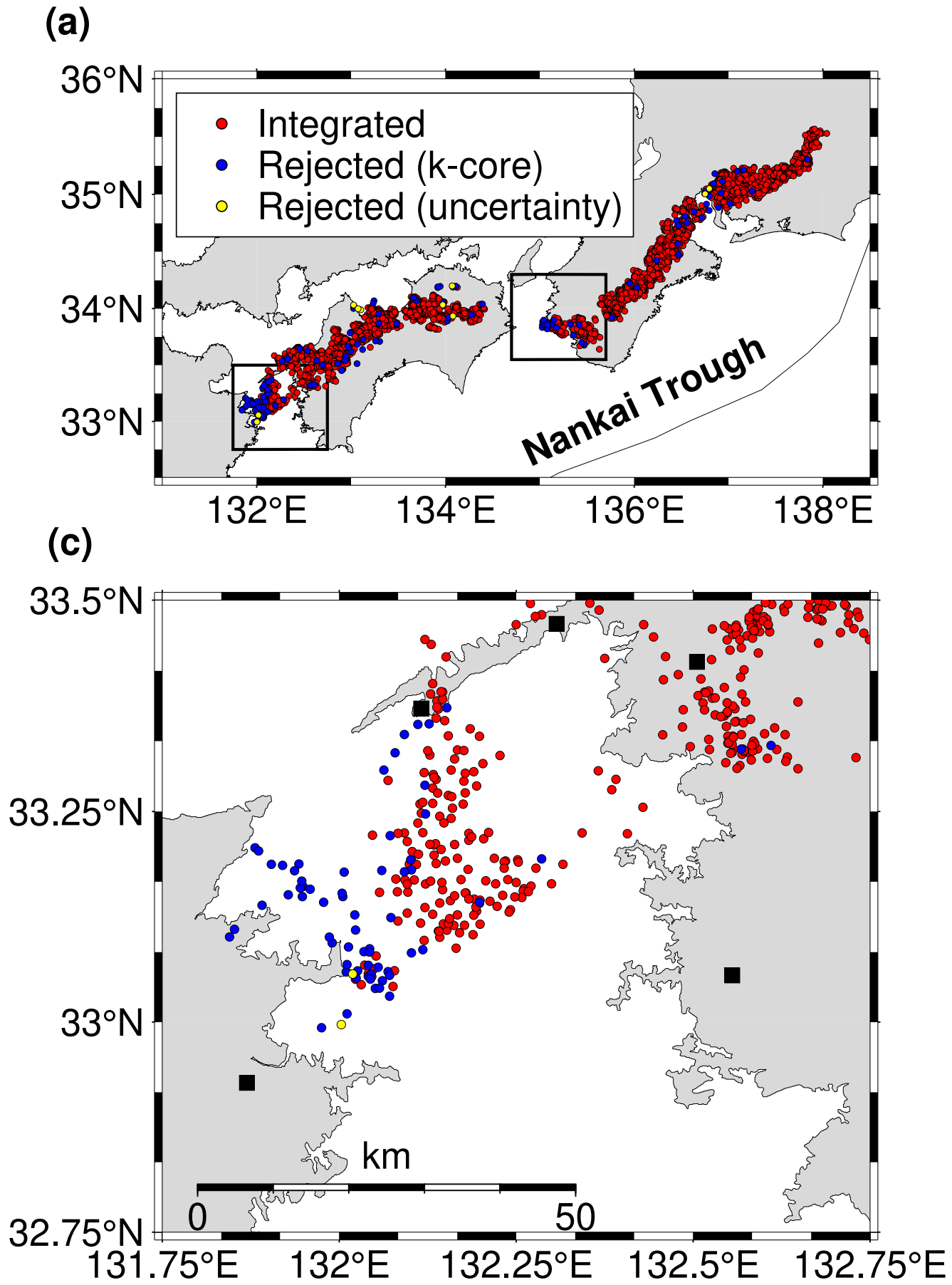

Fig. S6 Spatial distribution of tremor events accepted and rejected during the integration process. (a) Classification of events based on the network-based integration of single-station predictions. Red circles indicate accepted events that were successfully localized. Rejected events are categorized into those excluded by the uncertainty threshold (yellow circles) and those filtered out by the *k*-core algorithm (blue circles). **(b)** Distribution of the 94 seismic stations used in the integration. **(c, d)** Spatial distribution of events specifically rejected by the graph-based clustering. Black squares represent the locations of stations involved in these rejected predictions.

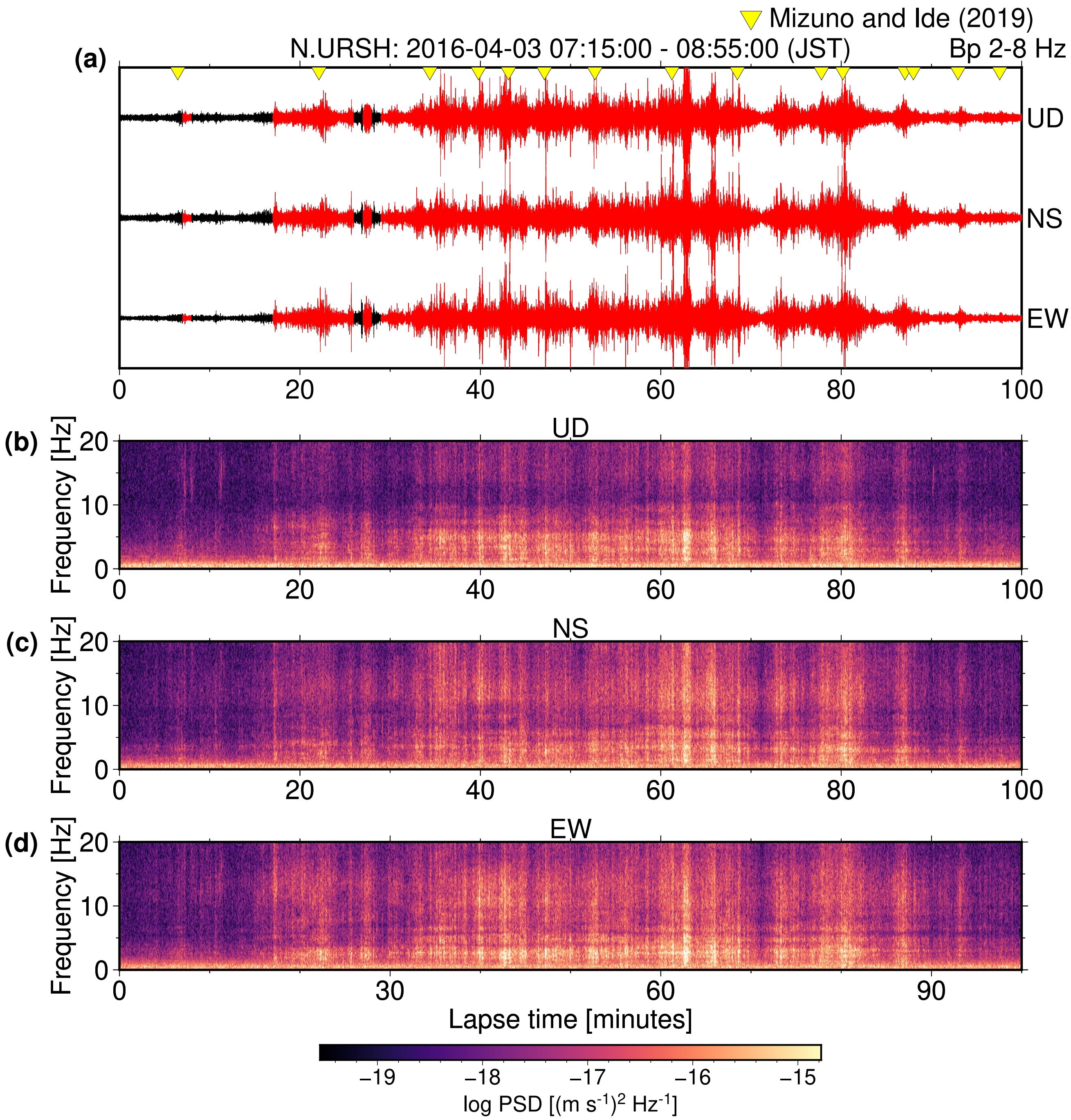

Fig. S7 Waveforms and spectrograms of tremors detected by the multi-station integration framework. (a) Bandpass-filtered (2–8 Hz) three-component velocity waveforms starting at 07:15 JST on 3 April 2016. Yellow triangles indicate tremor events listed in the Mizuno and Ide [2] catalog and red segments denote tremor detections through the integration process. **(b–d)** Corresponding spectrograms: **(b)** east–west (EW) component, **(c)** north–south (NS) component, and **(d)** vertical (UD) component.

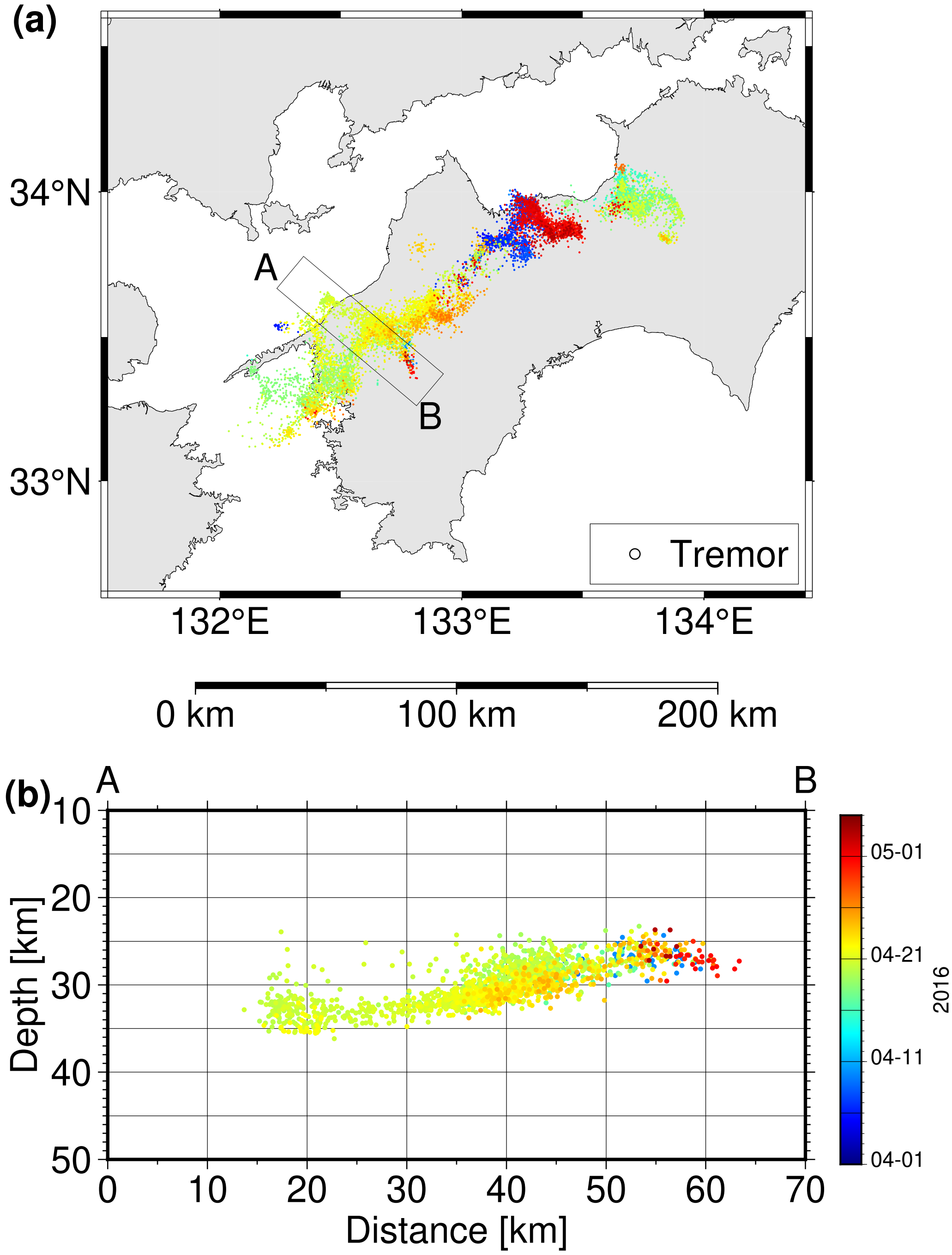

Fig. S8 Tremor epicentral and depth distributions obtained using the proposed location method for western Shikoku. **(a)** Map view of tremor epicenters. **(b)** Cross section of tremors located within the black rectangle shown in **(a)**. Dots indicate the located tremor hypocenters, with colors representing the temporal sequence of events.

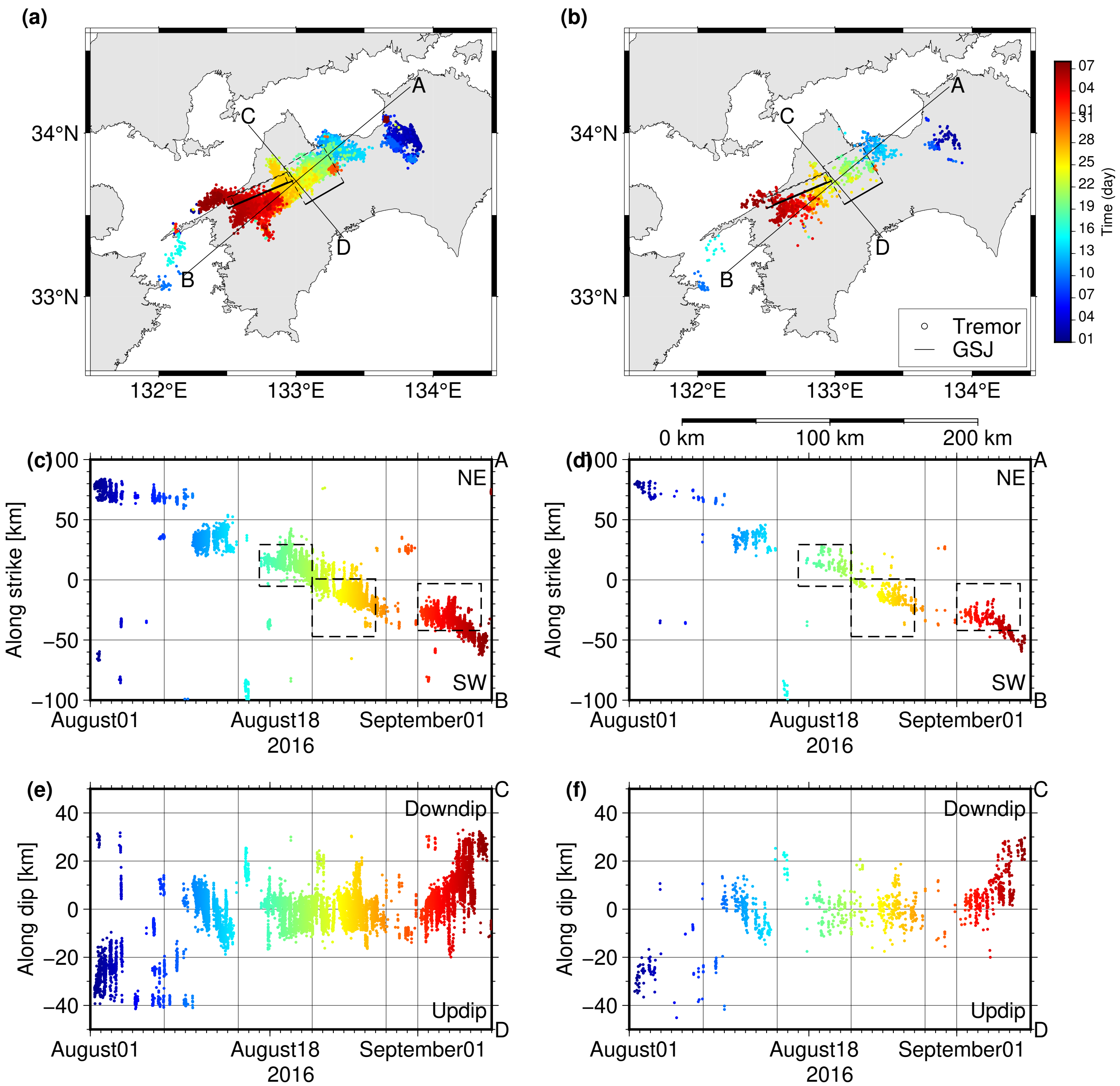

Fig. S9 High-resolution tremor migrations compared with existing catalogs. Comparison of the spatiotemporal evolution of tremor from 1 August to 8 September 2016 in **(a, c, e)** this study and **(b, d, f)** the Mizuno and Ide [2] catalog. **(a, b)** Map views of tremor epicenters. Black dashed rectangles indicate SSEs reported by GSJ[3,4]. **(c, d)** Spatiotemporal evolution along strike (profile A–B). **(e, f)** Cross-sectional views along the dip direction (profile C–D). In **(c–d)**, black dotted rectangles correspond to the SSEs from GSJ in **(a, b)**. In all panels, the color gradient represents the temporal progression of tremor activity.

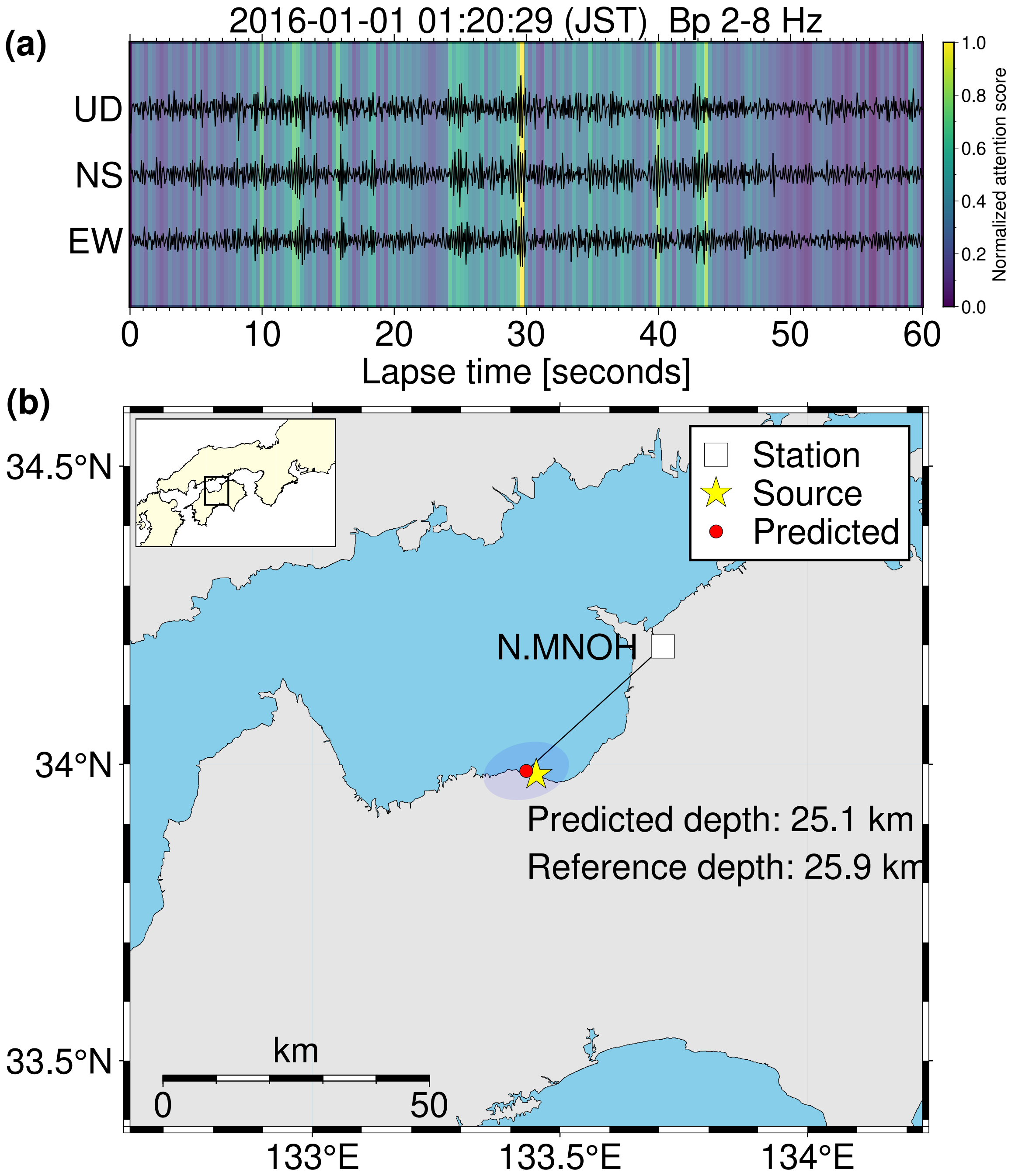

Fig. S10 Visualization of TremorViT attention rollout. (a) Three-component velocity waveforms (2–8 Hz) overlaid with normalized attention rollout scores at station N.MNOH starting at 01:20:29 on 1 January 2016. High scores highlight the temporal segments most influential to the model's prediction. **(b)** Comparison between predicted and cataloged epicenters. The red circle and blue ellipse denote the TremorViT-predicted epicenter and its 95% confidence interval, respectively. The yellow star represents the cataloged epicenter. The white squares indicate the station location. The concentration of high attention scores on tremor signal segments demonstrates that the model effectively identifies and prioritizes physically relevant waveform features.

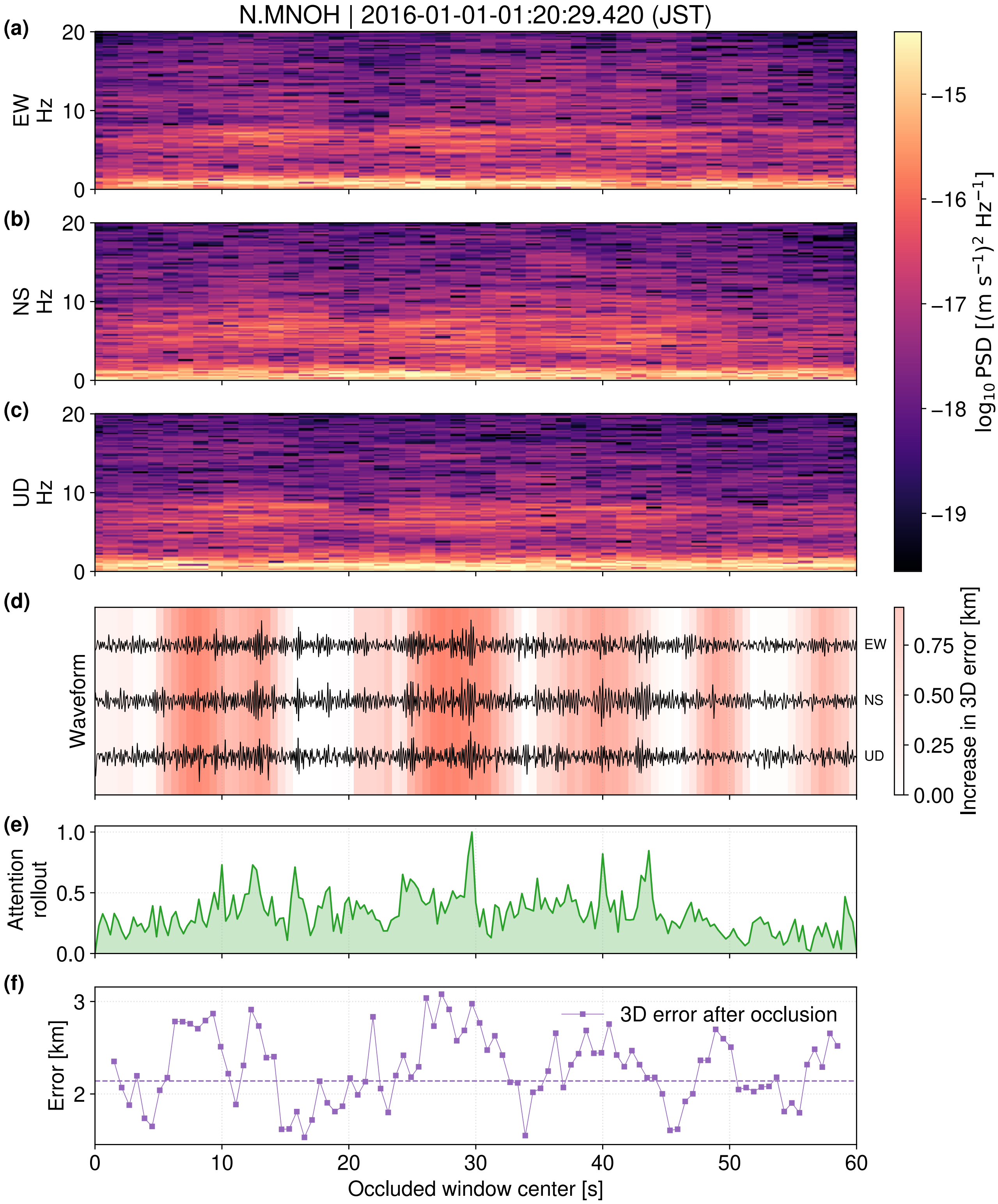

Fig. S11 Example of waveform features, attention weights, and occlusion sensitivity analysis at station N.MNOH (01:20:29.420 JST, 1 January 2016). (a–c) Spectrograms ($\log_{10}$ amplitude) of the bandpass-filtered (2–8 Hz) three-component velocity waveforms for the east–west (EW), north–south (NS), and vertical (UD) components, respectively. **(d)** Corresponding three-component waveforms. Red shaded regions indicate segments where occlusion causes larger hypocentral error,

highlighting waveform portions that strongly influence the model prediction. **(e)** Attention rollout values, showing the temporal importance assigned by the model. **(f)** Changes in hypocentral error caused by waveform occlusion (3 s windows shifted every 0.6 s). Larger errors indicate higher sensitivity to the masked segments. The dotted lines represent the original source-location errors.

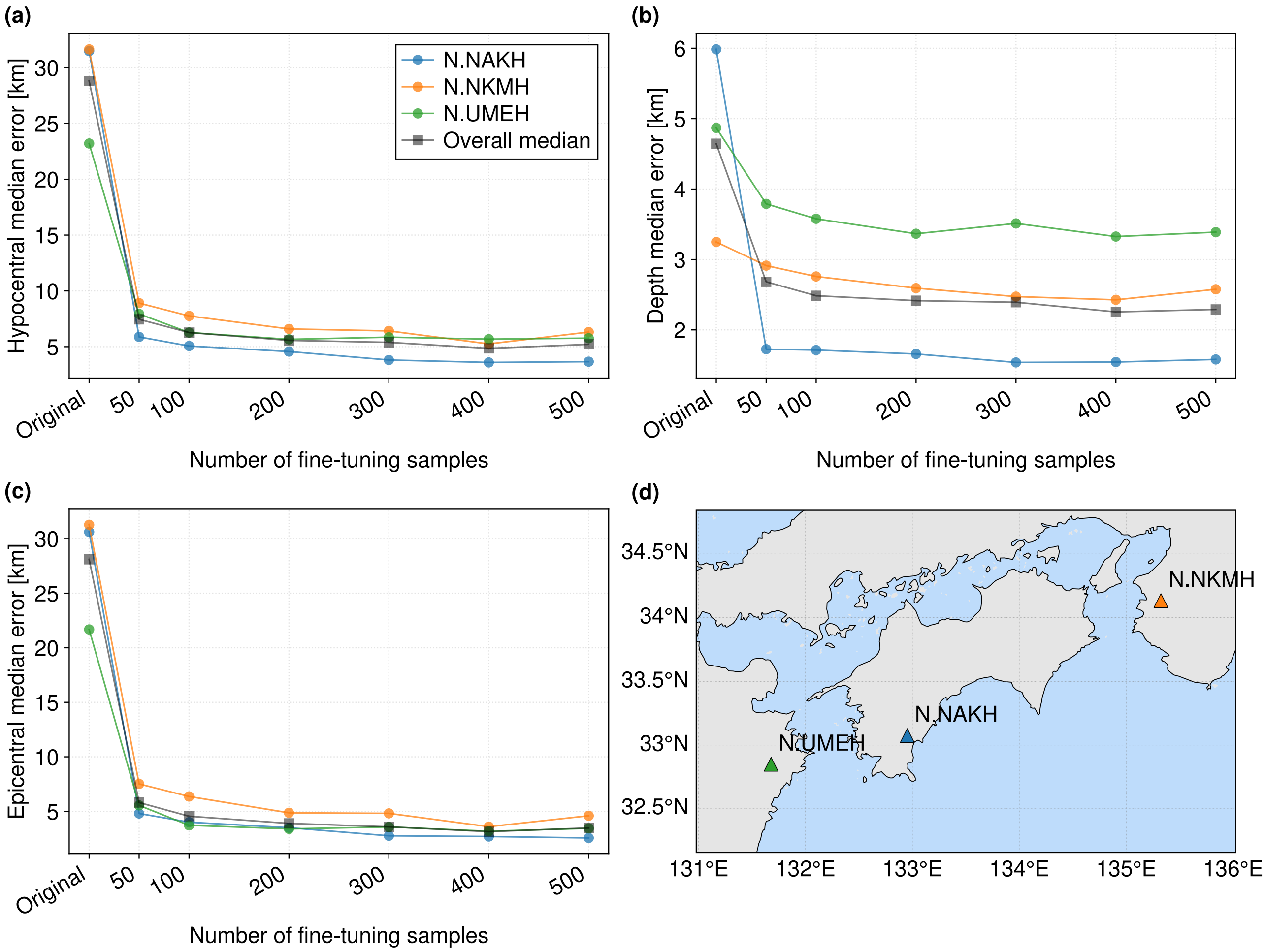

Fig. S12 Results of fine-tuning using stations not included in the training dataset. (a) Relationship between the number of training samples used for fine-tuning and the median hypocentral distance error. **(b)** Median depth error. **(c)** Median epicentral distance error. **(d)** Spatial distribution of the stations used for fine-tuning.

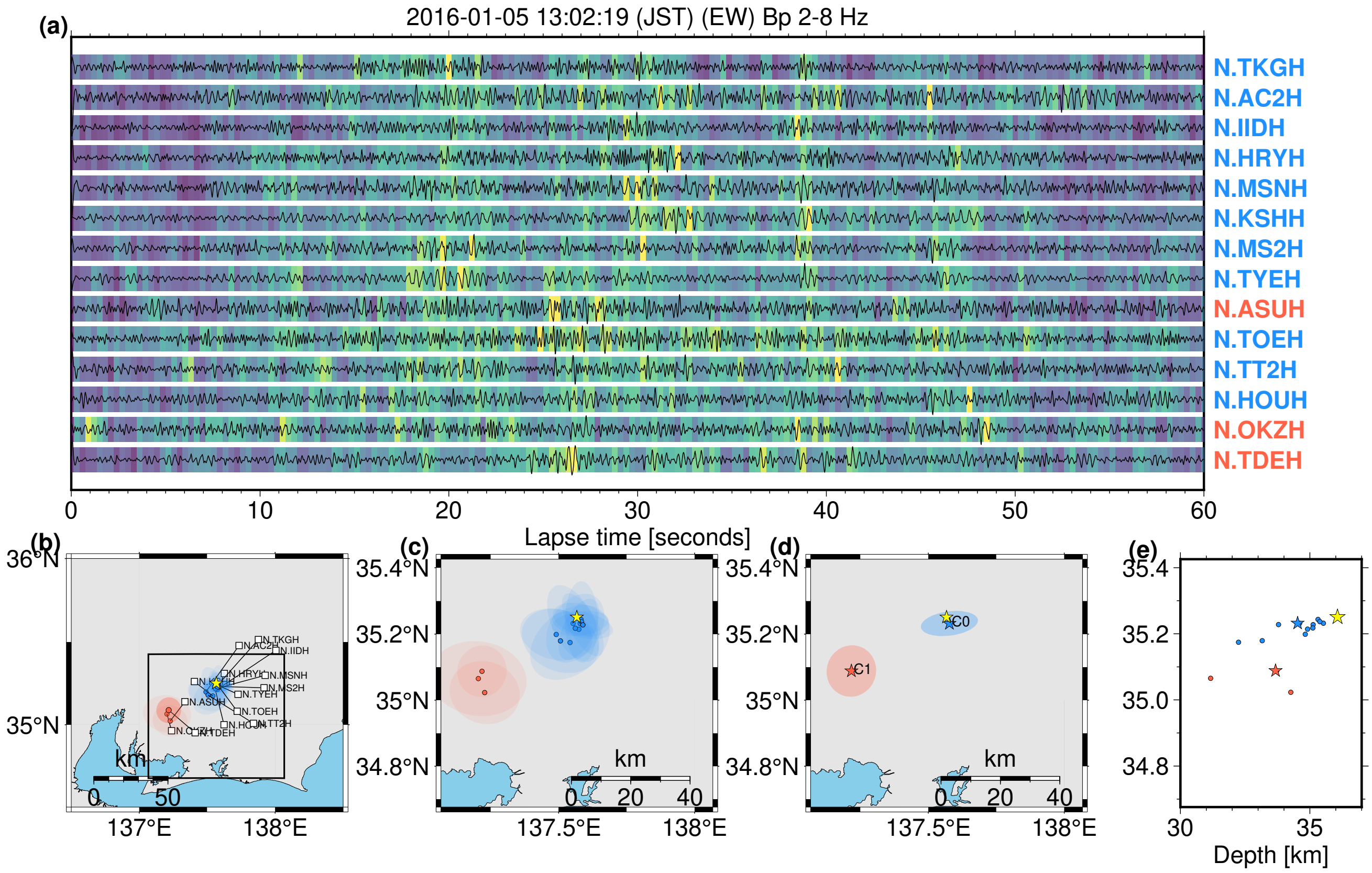

Fig. S13 Multi-cluster tremor detection through the integration of single-station predictions. (a) Bandpass-filtered (2–8 Hz) EW-component velocity waveforms at various stations starting at 13:02:19 on 5 January 2016. Overlaid scores indicate normalized attention rollout. Station names are color-coded (red and blue) by detected clusters. The theoretical S-wave arrival time is fixed at 30 s (V_S = 3.5 km/s). **(b)** Regional epicenter map. Circles and ellipses represent TremorViT predictions and 95% confidence intervals, respectively. The yellow star denotes a cataloged epicenter, and white squares indicate station locations. A rectangle indicates the enlarged region shown in **(c, d)**. **(c)** Single-station level epicenter distribution. **(d)** Final integrated epicenters for each cluster. **(e)** Depth distributions for the two clusters. Colored stars represent the final integrated depths. Circles denote single station-level depth predictions.

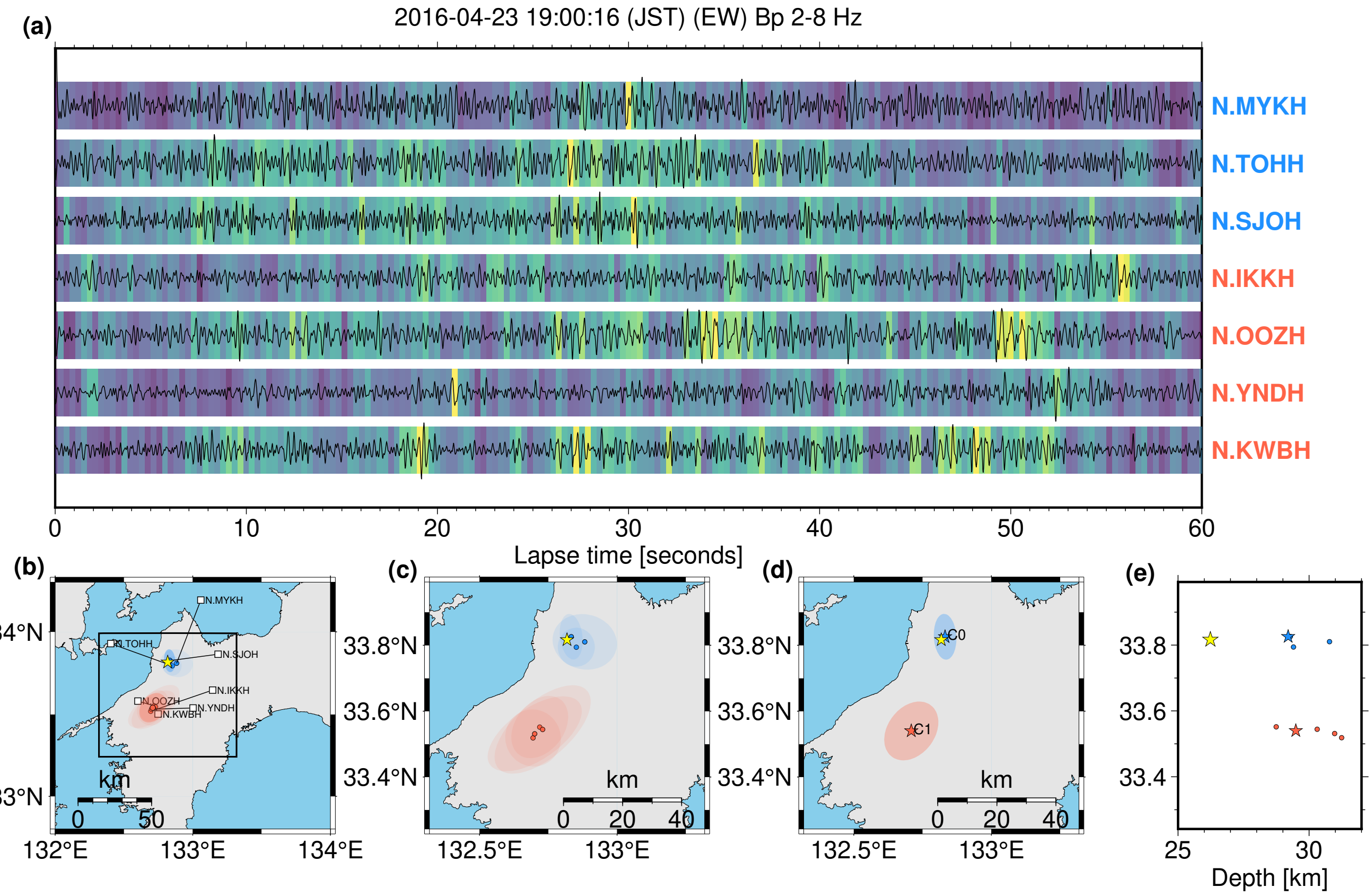

Fig. S14 Multi-cluster tremor detection through the integration of single-station predictions. As in Fig. S10, but starting at 19:00:16 JST on 23 April 2016.

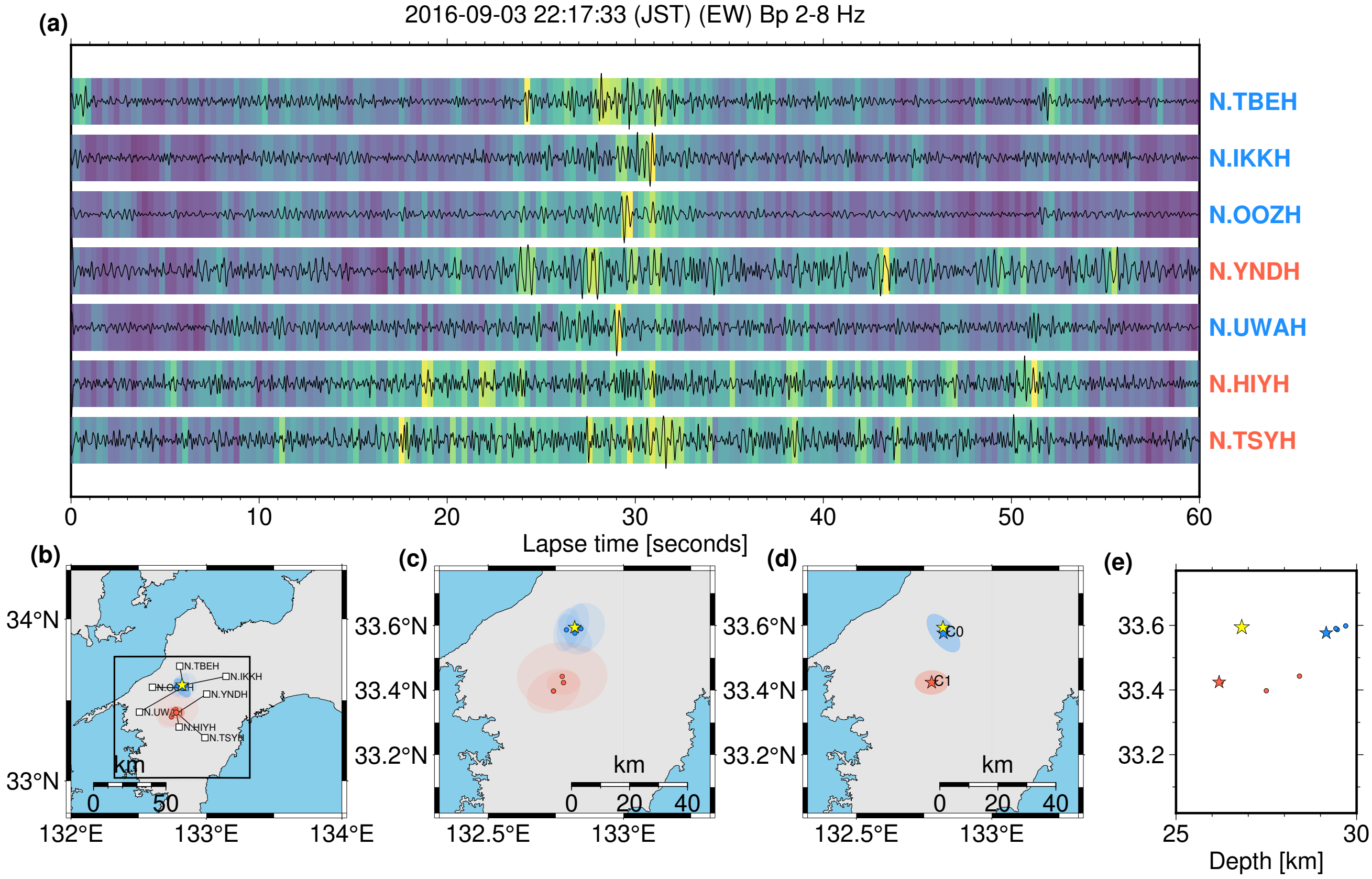

Fig. S15 Multi-cluster tremor detection through the integration of single-station predictions. As in Fig. S10, but starting at 22:17:33 JST on 3 September 2016.

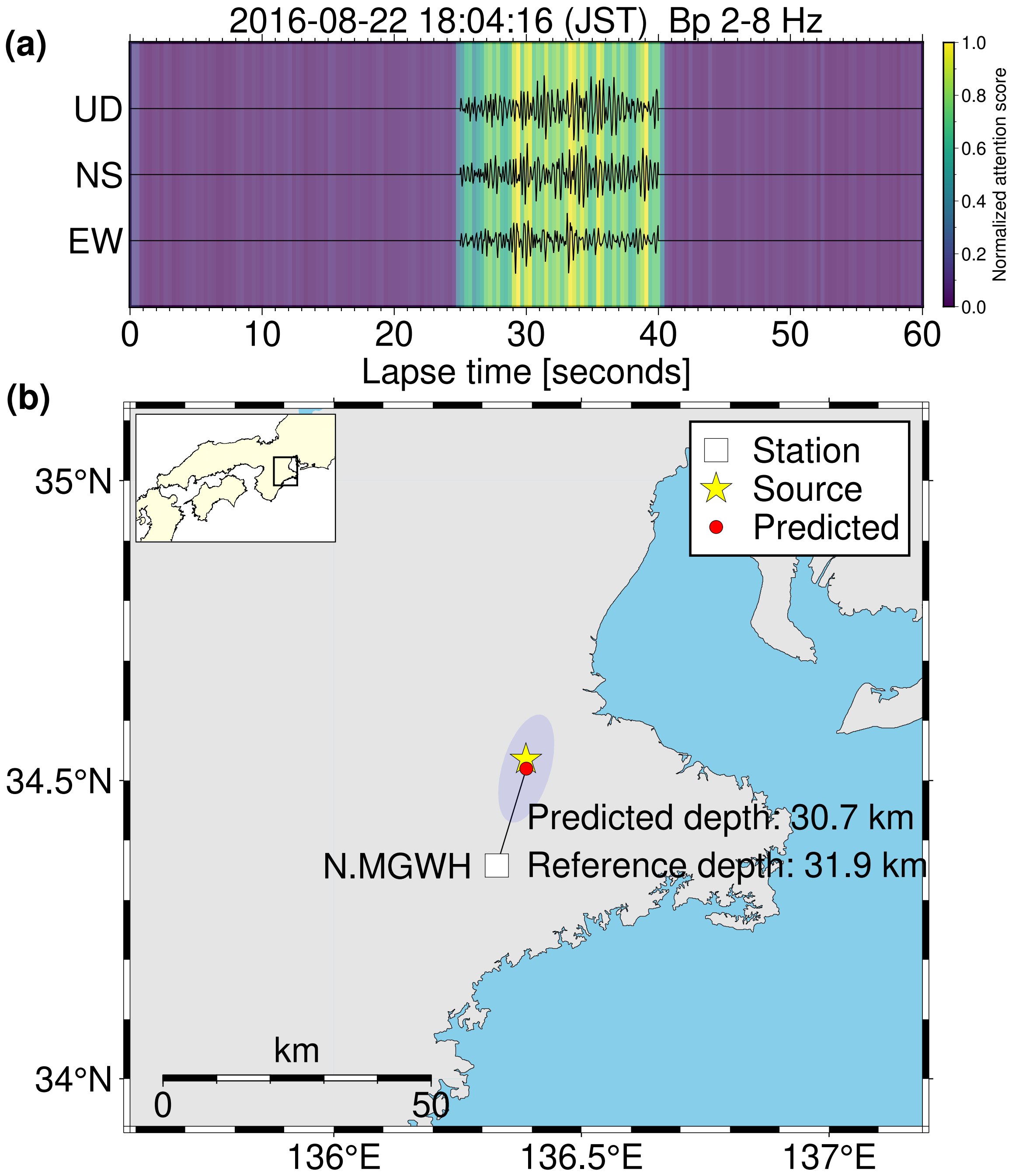

Fig. S16 Visualization of TremorViT attention rollout with masked waveform segments. (a) Three-component velocity waveforms (2–8 Hz) at station N.MGWH starting at 18:04:16 on 12 August 2016, overlaid with normalized attention rollout scores. Waveform amplitudes were masked to zero during the intervals 0–25 s and 40–60 s. High attention scores highlight the temporal segments most influential to the model's prediction. **(b)** Comparison between predicted and cataloged epicenters. The red circle and blue ellipse denote the TremorViT-predicted epicenter and its 95% confidence interval, respectively. The yellow star represents the cataloged epicenter. White squares indicate station locations. The concentration of high attention scores on tremor signal segments demonstrates that the model effectively identifies and prioritizes physically relevant waveform features.

References

1. Defazio, A. & Mishchenko, K. Learning-Rate-Free Learning by D-Adaptation. in *Proceedings of the 40th International Conference on Machine Learning* 7449–7479 (PMLR, 2023).

2. Mizuno, N. & Ide, S. Development of a modified envelope correlation method based on maximum-likelihood method and application to detecting and locating deep tectonic tremors in western Japan. *Earth Planets Space* **71**, 40 (2019).

3. Ochi T. *et al.* Short-term slow slip events in the Tokai area, the Kii Peninsula and the Shikoku District, Japan (from May to October 2016). *Rep. Coord. Comm. Earthq. Predict.* **97**, 242–253 (2017).

4. Ochi T. *et al.* Short-term slow slip events in the Tokai area, the Kii Peninsula and the Shikoku District, Japan (from November 2015 to April 2016). *Rep. Coord. Comm. Earthq. Predict.* **96**, 255–270 (2016).
